# Clinical Efficacy of Repeated Applications of Local Drug Delivery and Adjunctive Agents in Nonsurgical Periodontal Therapy: A Systematic Review

**DOI:** 10.3390/antibiotics10101178

**Published:** 2021-09-28

**Authors:** Oi Leng Tan, Syarida Hasnur Safii, Masfueh Razali

**Affiliations:** 1 Department of Restorative Dentistry, Faculty of Dentistry, Universiti Kebangsaan Malaysia, Jalan Raja Muda Abdul Aziz, Kuala Lumpur 50300, Malaysia; oileng1086@gmail.com; 2 Oral Health Division, Ministry of Health Malaysia, Putrajaya 62590, Malaysia; 3 Department of Restorative Dentistry, Faculty of Dentistry, University of Malaya, Kuala Lumpur 50603, Malaysia; syarida.safii@um.edu.my

**Keywords:** evidence-based dentistry, antibacterial agents, local anti-infective agents, periodontal pocket, periodontal debridement, periodontitis

## Abstract

The aim of this systematic review is to compare the clinical efficacy of repeated applications of local drug delivery and adjunctive agents (LDAs) in nonsurgical periodontal therapy (NSPT) compared to subgingival mechanical debridement (SMD) alone. The Cochrane Central Register of Controlled Trials, MEDLINE, PubMed, EMBASE, Web of Science, hand-searched literature and grey literature databases were searched for randomized controlled clinical trials (RCTs) with a minimum of 6-month follow-up. The outcomes of interest were changes in probing pocket depth and clinical attachment level as well as patient-centred outcomes. Of 1094 studies identified, 16 RCTs were included in the qualitative analysis. Across 11 different adjuncts analysed, only two studies utilizing minocycline gel/ointment and antimicrobial photodynamic therapy (aPDT) with indocyanine green photosensitizer had statistically significant differences in primary outcomes when compared to their control groups. Only one study on aPDT methylene blue 0.005% had compared single versus multiple applications against its control group. A mean range of 0.27–3.82 mm PD reduction and −0.09–2.82 mm CAL gain were observed with repeated LDA application. Considerable clinical heterogeneity and methodological flaws in the included studies preclude any definitive conclusions regarding the clinical efficacy of repeated LDA applications. Future RCTs with a direct comparison between single and repeated applications should be conducted to confirm or refute the clinical advantages of repeated LDA application in the nonsurgical management of periodontitis.

## 1. Introduction

The 2019 Global Burden of Disease Study ranked oral disorders as first in prevalence globally with 30% of the 3.48 billion people affected having some form of periodontal disease [1]. The ultimate endpoint of periodontal therapy is the prevention of tooth loss, which is linked to a decline in oral health-related quality of life [2,3]. Nonsurgical periodontal therapy (NSPT) has been proven to improve the quality of life related to oral health within a short time [4], and supportive periodontal therapy plays a crucial part in maintaining long-term therapeutic outcomes [5,6].

Most individuals affected with periodontitis respond well to mechanical debridement alone with long-term success [7]. The use of adjuncts may be pivotal in some cases that do not respond adequately to treatment, especially if surgical options are not possible [8,9]. The latest Clinical Practice Guideline developed by the European Federation of Periodontology recommended that locally administered antiseptics and antibiotics may be considered as an adjunct to subgingival instrumentation for treatment of Stage I–III periodontitis with consideration of its cost and availability of products [10]. These evidence-based recommendations have also been adopted by the British Society of Periodontology for clinical application in the United Kingdom dental community [11]. The American Association of Periodontology suggested the use of sustained- or controlled-release local delivery antimicrobial agents when there is the presence of a localized probing pocket depth (PD) of more than 4 mm and gingival inflammation following conventional therapies in the absence of anatomical defects [12], whereas the American Dental Association Clinical Practice Guidelines [13] recommended the use of doxycycline hyclate (DH) gel and minocycline (MINO) microspheres as an “expert opinion for” adjunctive use, which does not imply endorsement but signified that there was a lack of good evidence. The recommendation for chlorhexidine (CHX) chips and antimicrobial photodynamic therapy (aPDT) to be used as an adjunct in nonsurgical periodontal therapy was ‘weak’, albeit a with moderate level of certainty, and the authors proposed that the interventions should be implemented only after other alternatives have been considered.

There are various types of local adjuncts utilized in the treatment of periodontitis that include antimicrobials, probiotics and medical devices [10,14]. A medical device is described as “an instrument, apparatus, implement, machine, contrivance, implant, in vitro reagent, or other similar or related articles, including a component part or accessory” with the intent to alter the body’s function or structure without its chemical reaction and primary metabolism [15]. To date, hyaluronic acid and enamel matrix derivatives have been registered as medical devices under FDA, whereas, aPDT used in dentistry comprises of a chemical agent (photosensitizer dye) and a medical device (laser) component. Recently, biological mediators such as polypeptide growth factors have also been introduced for use as an adjunct in nonsurgical periodontal therapy [16,17].

Local adjuncts can be generally classified based on the duration of their availability within the periodontal pocket, being nonsustained-, sustained- or controlled-release delivery [18]. The highly concentrated active agents are expected to be retained within the periodontal pockets to eradicate causative bacteria, impede the formation of subgingival dental biofilm or aid in the early resolution of inflammation and promote wound healing with controlled-release devices having a longer duration (more than 24 h) of LDA retention compared with sustained-release devices (less than 24 h) [19].

The application of local adjuncts could minimize possible adverse effects and avoid the development of antibiotics resistance from systemic antimicrobials [20]. Previous systematic reviews (SRs) on local drug delivery and adjunctive agents (LDAs) [21,22,23,24,25,26,27,28] concluded that local adjuncts have additional clinical benefits up to 1.13 mm in PD reduction and 1.09 mm in clinical attachment level (CAL) gain compared to mechanical debridement alone. Nevertheless, different adjuncts demonstrate variable clinical efficacy according to their respective mechanism of actions [8]. A recent network meta-analysis (NMA) on LDAs [26] concluded that a single application of sulfonic/sulfuric acid gel and DH gel were the most effective in reducing PDs and gaining CALs in split-mouth and parallel study designs, respectively. Sulfonic/sulfuric acid gel demonstrated probable to definite clinical significance as an adjunct. The moderate certainty of evidence indicates that these adjuncts have probable superiority in the primary outcome measures over subgingival mechanical debridement (SMD) alone.

However, the effects of the frequency of LDA applications were rarely discussed in the systematic reviews. Previous SRs did not segregate the frequency of LDA application in their meta-analyses [21,22,23,24,25,27]. Furthermore, recent NMAs on the topic reported findings on single applications of LDAs so as to avoid heterogeneity in the analysis [26,29]. Repeated applications of LDAs are a valid clinical consideration in improving periodontal outcomes due to the issue of substantivity of the agents as well as the influence of elevated gingival crevicular fluid flow within the inflamed periodontal pocket [30]. To date, there is no carrier that can maintain substantivity for more than 1 week. Therefore, it is imperative to review the available evidence on repeated applications and provide recommendations to improve future research in this aspect/area. To the best of our knowledge, the current SR would be the first to address the clinical efficacy of repeated applications of current commercially available LDAs as an adjunct to nonsurgical mechanical debridement in NSPT. This topic would be relevant as it would influence the cost benefit analysis of the product and may guide clinicians to decide on which LDA to use. The present systematic review was designed to fulfil the focus question: “In systematically healthy adult patients diagnosed with periodontitis, what is the most clinically efficient repeated application of LDA with SMD used to treat residual pockets, compared with SMD alone or SMD with placebo, in relation to clinical and patient-centered outcomes?”

## 2. Methods

### 2.1. Review Protocol and Registration

The review protocol was submitted and registered in the International Prospective Register of Systematic Reviews database (Registration number CRD42020137115). The systematic review and NMA were carried out in accordance with the recommendations in the Preferred Reporting Items for Systematic Reviews and Meta-Analyses (PRISMA) statement [31] and the Cochrane Handbook for Intervention Reviews [32].

### 2.2. Inclusion and Exclusion Criteria

#### 2.2.1. Population

Human randomized controlled trials (RCTs) with either split-mouth or parallel arm study design with a 6-month minimum follow-up were included in the present review. The participants were diagnosed with periodontitis, not medically compromised and older than 18 years. The definition of periodontitis was based on the latest 2017 Classification of Periodontal and Peri-Implant Diseases and Conditions consensus workshop, which found no differences in etiology and pathophysiology to distinguish chronic and aggressive periodontitis as separate disease entities [33].

#### 2.2.2. Interventions

The test groups included the use of LDAs with SMD. The LDA must be administered as an adjunct to SMD, be commercially available LDAs with regulatory approval and have a main immunological, metabolic or pharmacological mechanism of action within the periodontal pocket. The sites considered should not have had any surgical intervention prior to LDA application. The studies were considered for inclusion if the LDA application was repeated more than once [34]. The studies that used experimental, discontinued and/or banned LDAs, single application LDAs and systemic adjuncts were excluded.

#### 2.2.3. Comparisons

The control groups received SMD alone or SMD with placebo. The definition of SMD in this review includes “scaling and root planing (SRP)” and “ultrasonic scaling (U/S)” as both techniques are capable in removing calculi [35].

#### 2.2.4. Outcomes

Primary outcomes were PPDs and CALs. The secondary clinical and patient-based outcome measures were bleeding on probing (BOP), patient-related outcome measures and postoperative adverse effects associated with LDA.

### 2.3. Search Methods

The search strategies are accessible in Supplementary Material File S1. The search was conducted in the following electronic databases: Cochrane Central Register of Controlled Trials, PubMed, MEDLINE, EMBASE and Web of Science. Conference abstracts, ProQuest Dissertations and Theses Global, ClinicalTrials.gov and the World Health Organization International Clinical Trials Registry Platform were searched for grey literature. Hand search of relevant journals within 5 years and the references of all eligible reviews was conducted for any additional studies. The search was performed up to 31 July 2021 with no limitation in language or publication status.

### 2.4. Study Selection and Data Extraction

Two reviewers (OLT and MR) independently screened and selected eligible titles and abstracts by applying the predefined inclusion and exclusion criteria before assessing the suitability of each full-text article. Cohen’s kappa (κ) was calculated for interrater reliability. Discrepancies between reviewers were deliberated until a consensus was made, and another reviewer (SHS) was consulted when the disagreement persisted. A standardized data extraction form was used to capture details on study design, general study characteristics, participants, disease severity, treatments used, clinical outcome measures and adverse events. Study investigators and/or corresponding authors were contacted with a request to provide further information through electronic mail when necessary. The studies were excluded if the authors failed to respond. In the case of duplicate publication, the article with more complete data and the larger sample size was selected.

### 2.5. Assessment of the Risk of Bias of Selected Studies

The Cochrane Collaboration’s Risk of Bias tool (RoB 2.0) [36] was used for the quality assessment of the included studies. The instrument comprises the five domains of bias: (1) randomization process, (2) measurement of the outcomes, (3) deviations from intended interventions, (4) selection of the reported result, and (5) missing outcome data. Each domain was assessed as high risk, some concerns or low risk. An overall bias would be regarded as low risk if all domains had low risk of bias, some concerns if some domains had some concerns in risk of bias, and high risk if 1 or more domains had been evaluated to be of high risk of bias.

### 2.6. Data Analyses

The primary clinical outcome variables analyzed were the mean PD and CAL changes from baseline to post-treatment. When no information on standard deviation (SD) or standard error (SE) was provided, SD was calculated from confidence intervals, t-values or *p*-values [32]. The correlation coefficient (ρ) value was set to 0.25 for statistical imputation as previously recommended by Lesaffre et al. [37] and Su and Tu [38] if data was not available. For the purpose of results interpretation, a mean difference (MD) of 0 was chosen as the threshold for statistical significance.

## 3. Results

**Figure 1:**
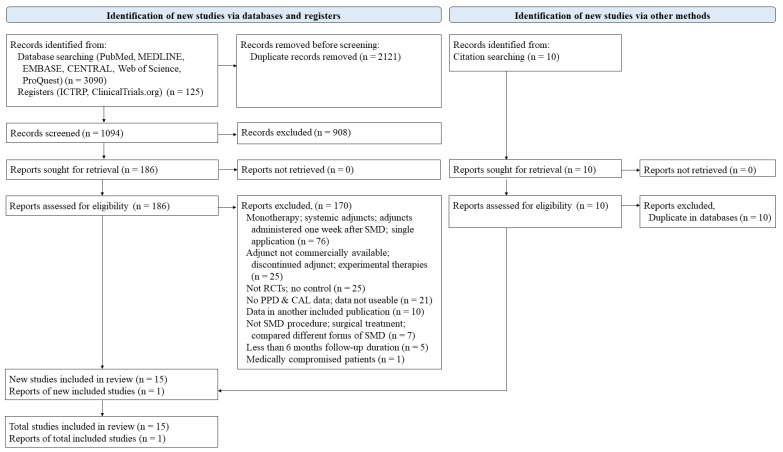
PRISMA flow diagram of the study selection process.

The initial screening identified 1094 studies from the literature search. Among them, 186 studies were chosen for full-text assessment after title and abstract screening (κ = 0.78, 95% CI 0.61 to 0.95). Only 16 studies met the eligibility criteria for NMA (κ = 0.78, 95% CI 0.69 to 0.87). The PRISMA flow diagram can be seen in Figure 1 and the list of full-text studies excluded, with reasons, is presented in Supplementary Material Table S1.

### 3.1. Study Characteristics

Nine parallel [39,40,41,42,43,44,45,46,47] and seven split-mouth [48,49,50,51,52,53,54] design trials were included with a total of 652 participants, within an age range between 20 and 82 years (Table 1). All of the studies were reported in English and conducted in fourteen countries from four continents. The selected studies were published as journal articles between 1999 and 2021, with the exception of a master’s thesis [45]. The duration of the included studies ranged between 6 months and 36 months, and 62.5% of the studies had less than 12 months follow-up [45,46,47,48,49,50,51,52,53,54]. Most trials were conducted in a university setting, except for two that involved private practices [44,50]. Two trials were multicentered [43,44] and six of the studies were commercially supported [39,40,41,45,49,50].

The 11 types of local adjuncts utilized were: (1) aPDT methylene blue (MB) 0.005% [50], (2) aPDT MB 1% [51], (3) aPDT indocyanine green (ICG) [52], (4) aPDT phenothiazine chloride [39,40], (5) aPDT toluidine blue O (TBO) [53], (6) chloramine gel [41], (7) CHX chip [46,47,48,49], (8) DH gel [44], (9) MINO gel [43,45], (10) MINO microspheres [42], and (11) povidone-iodine (PVP-I) subgingival irrigation [54]. Only four studies had more than 50 participants [42,43,44,45]. Clinical heterogeneity can be seen in the duration of studies, number of sites involved, frequency of local adjunct application and the control group used.

### 3.2. Risk of Bias within the Selected Studies

**Figure 2:**
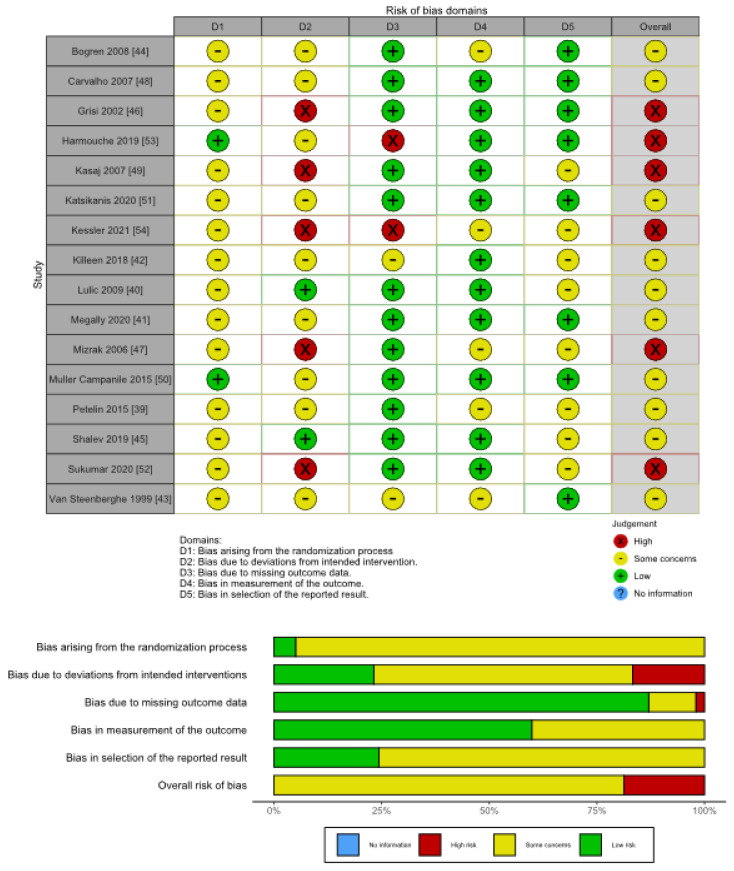
Risk of bias summary of included trials using RoB 2.0.

Risk of bias of the majority of the studies was scored as some concerns (62.5%; Figure 2). Six studies had a high risk of bias [46,47,49,52,53,54] and none of the studies had a low risk of bias score. Imperative data needed to measure the quality parameters were often incomplete or not reported. Bias that arose due to deviations from the intended interventions (effect of assignment to intervention) had the most serious issue in the methodology as most studies were single-blinded and did not utilize intention-to-treat analysis. Judgment of each risk of bias item for each included study according ROB2 can be found in Supplementary Material Table S2. Due to the high degree of clinical diversity or heterogeneity in the treatments evaluated, especially with regards to the frequency of applications, quantitative analysis was not conducted as it was considered inappropriate [55].

### 3.3. Primary Outcomes

#### PPD and CAL Changes

Greater mean PD reduction favoring the intervention group was seen in nine studies [40,41,43,44,45,47,48,49,52], whereas more CAL gain was seen with adjunct use in nice studies [40,41,43,44,45,47,49,50,52]. There were no significant statistical differences between intervention groups for both primary outcomes in the forementioned studies with the exception of two studies that utilized MINO ointment [43] and aPDT ICG [52] in which the authors found significant improvement in clinical and microbiological variables (Table 2). Interestingly, loss of CAL was observed in a study that utilized aPDT PC as an adjunct at the end of a 12-month observation period in both intervention groups but with lesser extent in the test group, and they were not statistically significant [40]. The authors commented that although mean PD and CAL no longer differed between intervention groups after 12 months, repeated applications were recommended to be used during supportive therapy [40]. Direct comparison between single vs. repeated LDA application was only found in one study with an additional mean 0.60 ± 2.00 mm PD reduction and 0.80 ± 3.23 mm CAL gain from an extra application of aPDT MB 0.005% [50].

### 3.4. Secondary Outcomes

#### BOP, Patient-Related Outcome Measures, and Postoperative Adverse Effects Associated with LDA

Changes in BOP were reported in most studies (93.3%) except for Killeen et al. [42] and Kessler et al. [54], which had their data presented in graph form instead of in text. However, four separate gingival bleeding indices were described, and only six studies specified the index used [41,43,46,47,51,54] (Table 3). There was a significant reduction in BOP in aPDT studies that used ICG [52] and PC [39] as photosensitizers compared to the control sites, whereas BOP was found to be only significantly reduced in the control group of a CHX chip adjunct study [46]. Greater BOP reduction was also observed in repeated aPDT MB 0.005% application compared to a single adjunct application (48% vs. 22%) [50].

Four studies did not report information on the presence of complications [39,40,45,47] whilst seven studies noted no adverse events post-treatment from the total adverse events reported [41,42,44,48,51,52,53,54] (Table 3). One CHX chip study [48] reported no adverse events, although two patients had their CHX chip dislodged, and a replacement was reinserted after 2 days. Based on a visual analogue scale (VAS) assessing root hypersensitivity, the chloramine gel study described a slight increase in the VAS score in both control and test groups [41]. The participants commented on smelling chlorine post-treatment with chloramine gel, but they perceived less pain post-treatment in the test group compared to the control; however, the difference was not significant.

Only two studies provided information on the cost-effectiveness of LDA. When the time needed for delivering treatment was compared, an average of 7−9 min per tooth was spent on the control group whereas 45−60 min was used to treat five to seven teeth with adjunctive application of aPDT [50]. On the other hand, chloramine gel required slightly more time to deliver the allocated treatment in the test group compared to the control group (372 ± 174 s vs. 238 ± 176 s, *p* = 0.015) [41].

## 4. Discussion

### 4.1. Summary of Findings

The present SR evaluated the clinical efficacy of LDAs against SMD alone when utilized in NSPT with at least 6 months follow-up. Although some studies concluded that repeated LDA applications offered more advantages to the overall outcomes of periodontal therapy [40,43,45,47,49,50,52,53], our findings found limited clinical improvements compared with SMD alone whereby the majority of the studies exhibited no significant differences between SMD alone and SMD with an adjunct. Since treatment with SMD alone would produce substantial improvement in PD and CAL [7,25], achieving an additional improvement with adjunctive application that is statistically significant can be challenging. Furthermore, the constant flow of gingival crevicular fluid into periodontal pockets may reduce the substantivity of the adjunct, thus rendering it less effective [30].

Our review demonstrated that different LDAs have different clinical efficacies, even with the use of a similar device such as aPDT. Based on the results of our study, different types and concentrations of photosensitizer dyes in aPDT may not exhibit the same clinical improvements, and this is supported by a recent SR that had shown favorable effect for ICG and PC [56]. Each dye varies in activation wavelength and may exhibit different clinical effectiveness with their bactericidal properties [57]. Commercially available diode laser systems that are advertised for aPDT use also have various photosensitizer dyes with different wavelength settings.

The present SR reported PD reduction and CAL gain of a mean range of 0.27–3.82 mm (vs. 0.07–2.90 mm) and −0.09–2.82 mm (vs. −0.20–3.00 mm), respectively, with repeated LDA application compared to SMD alone. On the whole, the repeated use of LDAs seems to be lacking in statistical and clinical significance with all included studies demonstrating some concerns and a high risk of bias. A minimum of 2 mm change in attachment level was proposed in a Cochrane review for a minimally important clinical difference in effectiveness [58]. Minor to moderate post-treatment adverse events were reported in 4 studies (25%) utilizing various adjuncts. On this account, the LDAs are considered safe for their intended use in clinical practice.

The definitions of the severity of periodontitis in the included studies were in alignment with localized and generalized Stage II or III and Grade B or C (for participants modified by smoking) periodontitis [59] based on the latest classification. However, specific reclassification of the disease was impossible due to the lack of information reported in the studies.

The intention of selecting only current commercially available LDAs for this study was to enable clinicians to make a practical decision based on what is available in the market with standardized compositions of a particular adjunct. Even though studies reporting experimental LDAs are important, we decided not to include them in this review as they did not fit in our objective and the products are not readily available to be used by clinicians. A regulatory-approved adjunct, available in the market, is guaranteed to be safe and effective for its intended use. Although there are plenty of different products used as adjuncts in treating periodontitis, such as probiotics, enamel matrix derivatives and hyaluronic acid, unfortunately these studies did not meet our inclusion criteria because they had short-term follow-ups.

### 4.2. Comparison of Findings to Other Reviews

The present study would be the first SR to report findings on repeated LDA application of various commercially available local antibiotics, antiseptics and medical devices. Previous pairwise SRs [21,22,23,24,25] reported PD reduction in a mean range of 0.32–0.41 mm and CAL gain in a mean range of −0.01–0.64 mm in a mixture of single and repeated LDA applications. The number of applications would be difficult to discuss as the products evaluated have a large variation; therefore, authors of a previous SR on adjunctive local antimicrobials did not conduct a subgroup analysis [24]. A recent NMA on locally delivered antimicrobial adjuncts during supportive care included single and repeated applications in their analysis and found that tetracycline fiber and CHX chip ranked the best with PD reductions of 0.64–0.65 mm and CAL gains of 0.31–0.60 mm in over 6 months [27]. However, the authors reported inconsistency in the observed effects, attributed to the different frequencies of aPDT application [27]. Evidence that supports repeated aPDT application in periodontal maintenance therapy remains inconclusive due to the lack of standardization in clinical protocols [34,56].

Another SR on local adjuvant therapies used to treat periodontitis Grade C severity found that simvastatin gel, alendronate gel and metronidazole gel showed some clinical benefits, but the the results were inconclusive and “off-label” products were included in the analysis [60]. Their clinical results differ from our previous [26] and current review as we segregated the analysis based on the frequency of LDA application as well as excluded discontinued and experimental adjuncts to cater to the current LDA market for the benefit of clinicians. Direct comparison between single and repeated LDA applications is rarely explored and was only reported in two RCTs from our search [50,61], in which the authors found some added benefits in the repeated use compared with the single application of their studied adjunct. The present SR suggests that it may be inappropriate to make any definitive conclusions with regards to the clinical efficacy of repeated LDA applications since there was considerable heterogeneity in the methodology.

This SR included a study on a new antiseptic gel that consists of amino acids and 0.95% sodium hypochlorite solution named Perisolv^®^ (Regedent AG, Zürich, Switzerland). The local agent was supposedly designed to enhance mechanical debridement by the chemical reaction between the amino acids and sodium hypochlorite, which forms chloramines that have a potent antimicrobial effect capable of dissolving degenerated tissue [62]. The study on PVP-I 10% subgingival irrigation did not find any additional benefits with use of the adjunct [54]. This is in line with what was previously reported on the inadequate reach of the irrigation solution into the subgingival crevice [63].

One of the few limitations of this review includes the insufficient number of studies and participants to conduct quantitative analysis. There was also a lack of direct comparison between single vs. repeated applications for the adjuncts to arrive to any conclusion regarding the clinical efficacy of applying LDA more than once. Moreover, it was not clearly stated whether the same sites had multiple applications or the adjunct was applied at a different site in the same patient at subsequent visits. Therefore, the true value of repeated applications cannot be objectively determined. Furthermore, a high number of studies presented methodological flaws in per-protocol data analysis and blinding, which resulted in some concerns in their risk of bias. The scarce research data on the frequency of absence of BOP and the formation of closed pockets, which ideally should be the desired clinical endpoints of treatment success [64], and the large heterogeneity of the bleeding indices and measurements used in the studies, which varied in mean values and percentages, made it difficult to standardize and summarize its effect.

## 5. Conclusions

Within the limitations of this SR, the following implications can be summarized:

- Repeated LDA application has an observed mean range of 0.27–3.82 mm PD reduction and −0.09–2.82 mm CAL gain.
- LDAs had limited clinical improvements compared with SMD alone whereby the majority of the studies exhibited no significant differences between SMD alone and SMD with an adjunct.
- Different LDAs have different clinical efficacy, even with the use of similar device such as aPDT. LDAs are considered safe for their intended use in clinical practice with minor to moderate post-treatment adverse events reported.
- The repeated use of LDAs lack statistical and clinical significance with all included studies demonstrating some concerns and high risk of bias. Although some studies have a purportedly minimum mean 2 mm change in attachment level post-treatment, most of the studies were single-blinded and did not utilize intention-to-treat analysis.

Although some clinical benefits were observed from repeated LDA application compared with SMD alone, considerable clinical heterogeneity and methodological disadvantages in the included studies preclude any definitive conclusions regarding the clinical efficacy of repeated LDA applications. Therefore, recommendations that more RCTs with direct comparison between single and repeated applications, as well as patient-centered outcomes, should be conducted to confirm or refute the clinical advantages of repeated LDA application in the nonsurgical management of periodontitis.

## Figures and Tables

**Table 1:** Study characteristics of the included studies based on adjuncts.

Adjunct Used.	Study Citation	Trial Design, Country	Centre, Setting	Disease Severity, Definition	Number of Smokers (%)	Age Mean ± SD (Range)		Sex Male/Female	
						Test	Control	Test	Control
Chlorhexidine chip	Carvalho et al., 2007 [48]	Split-mouth, USA	1, university	mild to moderate CP, Nonmolar sites with PD > 4 mm and BoP	9/26 (35)	54.5 (35–81)		11/15	
	Grisi et al., 2002 [46]	Parallel, Brazil	1, university	CP, PD ≥ 5 mm and BoP	20, NS	43.3 ± 5.9 (35–56)	41.1 ± 5.4 (37–54)	5/5	3/6
	Kasaj et al., 2007 [49]	Split-mouth, Germany	1, university	moderate to severe CP, PD ≥ 5 mm and BoP	20, NS	42.0 ± 5.6 (20–60)		7/13	
	Mizrak et al., 2006 [47]	Parallel, Turkey	1, university	CP, PD 5–8 mm and radiographic bone loss	34, NS	35 ± 8.5 (20 to 55)		22/12	
Doxycycline hyclate gel	Bogren et al., 2008 [44]	Parallel, Sweden and USA	3, university and private practice	moderate to advanced periodontitis, PD ≥ 5 mm	38/128 (29.7)	58 (34–77)	60 (35–82)	44/19	42/23
Minocycline gel/ointment	Shalev 2019 [45]	Parallel, USA	1, university	moderate to severe periodontitis, PD 5–9 mm and BoP	22/59 (37.3), Ex-users: 19/59 (32.2)	53.8 ± 11.3 (NR)	Sham: 53.8 ± 11.3 (NR) Vehicle: 50.8 ± 0.5 (NR)	10/10	24/15
	van Steenberghe et al., 1999 [43]	Parallel, Belgium, Sweden, UK, Netherlands	6, university	moderate to severe CP, Interproximal sites with PD ≥ 5 mm, CAL ≥ 3 mm and radiographic bone loss	104, NR	48 ± 7 (35–64)	44 ± 7 (34–61)	43/50	
Minocycline microspheres	Killeen et al., 2018 [42]	Parallel, USA	1, university	moderate to severe CP, Interproximal posterior sites with PD ≥ 5 mm and BoP	12/55 (21.8)	67.3 ± 10.5 (NR)	66.8 ± 12.1 (NR)	22/5	16/12
Chloramine gel	Megally et al., 2020 [41]	Parallel, Switzerland	1, university	CP, Distal site of first incisor or mesial site of second molar with PD ≥ 5 mm	7/32 (21.9)	61.7 ± 9.8 (NR)	62.1 ± 8.8 (NR)	11/5	10/6
Antimicrobial photodynamic therapy (Methylene blue 0.005%)	Müller Campanile et al., 2015 [50]	Split-mouth, Switzerland	1, private practice	CP, PD > 4 mm, CAL > 1 mm and BoP	12/27 (44.4)	62.8 (37–77)		14/13	
(Methylene blue 1%)	Katsikanis et al., 2020 [51]	Split-mouth, Greece	1, university	moderate to severe CP, PD ≥ 5 mm	7/21 (33.3)	48.2 ± 8.2 (NR)		8/13	
(Indocyanine green)	Sukumar et al., 2020 [52]	Split-mouth, India	1, university	CP, Min 3 bilateral mandibular posteriors with PD 4–6 mm and CAL	33, NS	38.6 ± 6.8 (NR)		22/8	
(Phenothiazine chloride)	Lulic et al., 2009 [40]	Parallel, Switzerland	1, university	CP, PD ≥ 5 mm ± BoP	2/10 (20)	56 (44–74)	52 (40–57)	3/2	4/1
	Petelin et al., 2015 [39]	Parallel, Slovenia	1, university	CP, Minimum 4 teeth with PD ≥ 4 mm per quadrant	27, NS	47 (36–59)	51 (42–64)	5/4	4/5
(Toluidine blue O)	Harmouche et al., 2019 [53]	Split-mouth, France	1, university	generalized severe CP, PD ≥ 5 mm, CAL > 5 mm and BoP	11/36 (30.6)	50.25 ± 5.98 (NR)		14/22	
Povidone- Iodine subgingival irrigation	Kessler et al., 2021 [54]	Split-mouth, Belgium	1, university	stage II to III, grade A to B periodontitis, PD ≥ 4 mm, CAL ≥ 3 mm and BoP	NR	51.8 (34–62)		9/8	

NR, not reported; SD, standard deviation; PPD, probing pocket depth; CAL, clinical attachment level; BOP, bleeding on probing; NS, nonsmokers.

**Table 2:** Intervention characteristics and outcomes data of the included studies based on adjuncts.

Study Citation, Follow-Up	Interventions	Area, Frequency of Application	Test Group				Control Group			
			Sample Size BL/End of Study	Pre-Intervention Clinical Parameters Mean ± SD	Post-Intervention Clinical Parameters Mean ± SD	Change in Clinical Parameters Mean ± SD	Sample Size BL/End of Study	Pre-Intervention Clinical Parameters Mean ± SD	Post-Intervention Clinical Parameters Mean ± SD	Change in Clinical Parameters Mean ± SD
Carvalho et al., 2007 [48], 9 m	Test: CHX chip (Periochip^®^) + SRP Control: SRP	PM, 3× (BL, 3 m, 6 m)	28/26	PPD: 5.90 ± 1.30 CAL: 6.10 ± 2.10 BOP: 96%	PPD: 4.70 ± 1.30 CAL: 5.20 ± 1.90 BOP: 54%	PPD: 1.20 ± 1.59 CAL: 0.90 ± 2.39 BOP: 42%	28/26	PPD: 5.60 ± 1.10 CAL: 5.30 ± 1.40 BOP: 100%	PPD: 4.50 ± 1.40 CAL: 4.40 ± 1.90 BOP: 46%	PPD: 1.10 ± 1.55 CAL: 0.90 ± 2.06 BOP: 54%
Grisi et al., 2002 [46], 9 m	Test: CHX chip (Periochip^®^) + SRP Control: SRP	PM, 3× (BL, 3 m, 6 m)	10/10	PPD: 5.20 ± 0.60 CAL: NR BOP: 87.8%	PPD: 3.00 0.80 CAL: NR BOP: 65.9%	PPD: 2.20 ± 0.70 CAL: 0.60 ± 0.70 * BOP: 22%	10/9	PPD: 5.20 ± 0.50 CAL: NR BOP: 79.1%	PPD: 2.90 ± 0.60 CAL: NR BOP: 43.6%	PPD: 2.40 ± 0.70 CAL: 1.00 ± 0.40 * BOP: 36% *
Kasaj et al., 2007 [49], 6 m	Test: CHX chip (Periochip^®^) + U/S Control: U/S	PM, 2× (BL, 3 m)	20/20	PPD: 6.20 ± 1.00 CAL: 6.90 ± 1.60 BOP: 71%	PPD: NR CAL: NR BOP: 29%	PPD: 2.20 ± 0.80 CAL: 1.90 ± 1.10 BOP: 42%	20/20	PPD: 6.30 ± 0.90 CAL: 7.20 ± 1.40 BOP: 67%	PPD: NR CAL: NR BOP: 58%	PPD: 0.70 ± 0.60 CAL: 0.60 ± 0.70 BOP: 9%
Mizrak et al., 2006 [47], 6 m	Test: CHX chip (Periochip^®^) + SRP Control: SRP	PM, 2× (BL, 3 m)	17/17	PPD: 6.94 ± 0.74 CAL: 7.50 ± 0.80 BOP: 1.70 ± 0.46	PPD: NR CAL: NR BOP: NR	PPD: 3.82 * CAL: 2.82 * BOP: 1.05	17/17	PPD: 6.05 ± 0.89 CAL: 7.00 ± 1.10 BOP: 1.74 ± 0.43	PPD: NR CAL: NR BOP: NR	PPD: 2.35 CAL: 1.64 BOP: 0.88
Bogren et al., 2008 [44], 36 m	Test: DH gel (Atridox^®^) + SRP Control: SRP	FM, 3× (BL, 1y, 2y)	63/60	PPD: 5.40 (95% CI 5.33–5.57) CAL: NR BOP: 51%	PPD: 4.20 (95% CI 4.04–4.45) CAL: NR BOP: 50%	PPD: 1.20 ± 1.67 CAL: 0.90 ± 2.26 BOP: 1%	65/64	PPD: 5.60 (95% CI 5.44–5.77) CAL: NR BOP: 56%	PPD: 4.50 (95% CI 4.29–4.74) CAL: NR BOP: 38%	PPD: 1.10 ± 1.97 CAL: 0.70 ± 2.10 BOP: 18%
Shalev 2019 [45], 9 m	Test: MINO gel (Periocline ^®^) + SRP Control: Placebo + SRP	FM, 4× (BL, 2 w, 1 m, 3 m, 6 m)	20/20	PPD: 5.68 ± SE 0.09 CAL: 4.87 ± SE 0.26 BOP: 95.46% ± SE 1.48%	PPD: 3.91 ± SE 0.13 CAL: 3.32 ± SE 0.26 BOP: 32.50% ± SE 3.71%	PPD: 1.76 ± 0.63 CAL: 1.56 ± 0.54 BOP: 63% ± 3.94% *	39/39	Sham: PPD: 5.82 ± SE 0.13 CAL: 5.33 ± 0.25 BOP: 92.51% ± SE 2.57% Vehicle: PPD: 5.65 ± SE 0.08 CAL: 4.79 ± SE 0.22 BOP: 95.71% ± SE 1.31%	Sham: PPD: 4.29 ± SE 0.18 CAL: 3.94 ± SE 0.31 BOP: 40.84% ± SE 5.28 Vehicle: PPD: 3.96 ± SE 0.16 CAL: 3.18 SE 0.30 BOP: 49.63% ± SE 4.57%	PPD: 1.59 ± 0.61 CAL: 1.42 ± 0.53 BOP: 49% ± 5.36% *
van Steenberghe et al., 1999 [43], 15 m	Test: MINO gel (Dentomycin^®^) + SRP Control: Placebo + SRP	PM, 7× (BL, 2 w, 1 m, 3 m, 6 m, 9 m, 12 m)	52/45	PPD: 6.50 CAL: NR BOP: 2.50	PPD: 4.60 CAL: NR BOP: 1.40	PPD: 1.90 ± 0.32 CAL: 0.90 ± 0.39 BOP: 1.1 ± 0.85	52/45	PPD: 6.30 CAL: NR BOP: 2.50	PPD: 5.10 CAL: NR BOP: 1.70	PPD: 1.20 ± 0.31 CAL: 0.50 ± 0.37 BOP: 0.8 ± 0.82
Killeen et al., 2018 [42], 24 m	Test: MINO microspheres (Arestin^®^) + SRP Control: SRP	PM, 4× (BL, 6 m, 12 m, 18 m)	27/23	PPD: 5.29 ± 0.62 CAL: 5.42 ± 0.65 BOP: NR	PPD: 4.14 ± 0.89 CAL: 4.36 ± 1.05 BOP: NR	PPD: 0.80 ± 0.90 CAL: 0.80 ± 0.90 BOP: NR	28/25	PPD: 5.48 ± 0.75 CAL: 5.81 ± 0.92 BOP: NR	PPD: 3.96 ± 0.73 CAL: 4.24 ± 0.66 BOP: NR	PPD: 1.00 ± 0.60 CAL: 1.00 ± 0.70 BOP: NR
Megally et al., 2020 [41], 12 m	Test: Chloramine gel (Perisolv^®^) + U/S Control: U/S	PM, 3× (BL, 4 m, 8 m)	16/16	PPD: 5.39 ± 0.62 CAL: NR BOP: 89%	PPD: 4.43 ± 1.07 CAL: NR BOP: 83%	PPD: 0.97 ± 1.09 * CAL: 1.02 ± 1.49 * BOP: 6%	16/16	PPD: 5.31 ± 0.58 CAL: NR BOP: 88%	PPD: 4.46 ± 1.19 CAL: NR BOP: 73%	PPD: 0.85 ± 1.13 * CAL: 0.82 ± 1.33 * BOP: 15%
Müller Campanile et al., 2015 [50], 6 m	Test: aPDT MB 0.005% (Periowave^TM^) + U/S Control: Sham + U/S Laser: 280 mW and 670 nm	PM, 2× (BL, 1 w)	28/27	PPD: 5.90 ± 0.90 CAL: 7.00 ± 1.60 BOP: 59.26%	PPD: 3.10 ± 1.00 CAL: 4.10 ± 1.60 BOP: 37.04%	PPD: 2.80 ± 1.17 CAL: 2.90 ± 1.96 BOP: 22%	28/27	PPD: 6.30 ± 1.50 CAL: 7.60 ± 2.00 BOP: 55.56%	PPD: 3.40 ± 1.50 CAL: 4.60 ± 2.20 BOP: 37.04%	PPD: 2.90 ± 1.84 CAL: 3.00 ± 2.58 BOP: 19%
Katsikanis et al., 2020 [51], 6 m	Test: aPDT MB 1% + SRP Control: SRP Laser: 350 mW and 670 nm	PM, 3× (48 h, 1 w, 2 w)	21/21	PPD: 4.76 ± 0.79 CAL: 5.49 ± 1.52 BOP: 79%	PPD: NR CAL: NR BOP: 15.9	PPD: 1.66 ± 1.02 CAL: 1.04 ± 1.19 BOP: 63%	21/21	PPD: 4.80 ± 0.76 CAL: 5.29 ± 1.17 BOP: 81.9%	PPD: NR CAL: NR BOP: 13.3%	PPD: 1.66 ± 1.14 CAL: 1.24 ± 1.35 BOP: 69%
Sukumar et al., 2020 [52], 6 m	Test: aPDT ICG 0.1% + SRP Control: SRP Laser: 800 mW and 810 nm	PM, 4× (BL, 1 w, 2w, 4w)	33/30	PPD: 5.93 ± 0.82 CAL: 5.73 ± 0.69 BOP: 2.0	PPD: 3.40 ± 0.56 CAL: 3.00 ± 0.91 BOP: 0.17 ± 0.37	PPD: 2.53 ± 0.87 * CAL: 2.73 ± 1.00 * BOP: 1.8 ± 0.37 *	33/30	PPD: 5.83 ± 0.64 CAL: 5.60 ± 0.72 BOP: 2.0	PPD: 3.80 ± 0.40 CAL: 3.70 ± 0.91 BOP: 0.6 ± 0.35	PPD: 2.03 ± 0.66 * CAL: 1.90 ± 1.01 * BOP: 1.4 ± 0.35 *
Lulic et al., 2009 [40], 12 m	Test: aPDT PC (HELBO^®^) + SRP Control: Sham + SRP Laser: 75 mW and 670 nm	FM, 5× (BL, 1 d, 2 d, 7 d, 14 d)	5/5	PPD: 6.08 ± 1.19 CAL: 6.70 ± 2.17 BOP: 97%	PPD: 5.81 ± 1.33 CAL: 6.79 ± 2.37 BOP: 77%	PPD: 0.27 ± 0.43 CAL: −0.09 ± 0.41 BOP: 20% *	5/5	PPD: 5.90 ± 0.71 CAL: 7.55 ± 1.73 BOP: 84%	PPD: 5.93 ± 0.49 CAL: 7.76 ± 1.66 BOP: 87%	PPD: 0.07 ± 0.61 CAL: −0.20 ± 0.61 BOP: −3%
Petelin et al., 2015 [39], 12 m	Test: aPDT PC (HELBO^®^) + U/S Control: U/S Laser: 60 mW and 660 nm	FM, 3× (BL, 3 d, 7 d)	9/9	PPD: 3.40 ± 0.20 CAL: 4.20 ± 0.30 BOP: 24.9% ± 2.8%	PPD: 2.90 ± 0.20 CAL: 3.70 ± 0.20 BOP: 9.4% ± 1.4%	PPD: 0.50 ± 0.24 CAL: 0.50 ± 0.32 BOP: 16% ± 2.8% *	9/9	PPD: 3.60 ± 0.20 CAL: 4.30 ± 0.30 BOP: 23% ± 2.8%	PPD: 3.00 ± 0.20 CAL: 3.70 ± 0.20 BOP: 12.2% ± 1.4%	PPD: 0.60 ± 0.24 CAL: 0.60 ± 0.32 BOP: 11% ± 2.8% *
Harmouche et al., 2019 [53], 6 m	Test: aPDT TBO (FotoSan^®^) + SRP Control: Sham + SRP Laser: 2 W and 628 nm	PM, 3× (BL, 1 w, 3 m)	36/28	PPD: 4.06 ± 1.71 CAL: 4.79 ± 2.07 BOP: 64.89%	PPD: 2.93 ± 1.42 CAL: 3.94 ± 1.99 BOP: 32.48%	PPD: 1.13 ± 1.93 CAL: 0.85 ± 2.49 BOP: 32%	36/28	PPD: 4.10 ± 1.72 CAL: 4.77 ± 2.06 BOP: 64.64%	PPD: 2.94 ± 1.43 CAL: 3.92 ± 1.93 BOP: 33.12%	PPD: 1.16 ± 1.94 CAL: 0.85 ± 2.45 BOP: 32%
Kessler et al., 2021 [54], 6 m	Test: PVP-I 10% (iso-Betadine, Dermal) + SRP Control: 0.9% NaCl + SRP	FM, 3× (BL, 3 m, 6 m)	17/22	PPD: 3.70 ± 0.90 CAL: 3.90 ± 0.90 BOP: 64% 26.9%	PPD: 2.50 ± 0.60 CAL: 3.00 ± 0.80 BOP: NR	PPD: 1.20 ± 0.95 CAL: 0.80 ± 1.04 BOP: NR	17/22	PPD: 3.70 ± 0.70 CAL: 3.90 ± 0.80 BOP: 56% 29.3%	PPD: 2.60 ± 50 CAL: 3.20 ± 0.70 BOP: NR	PPD: 1.10 ± 0.75 CAL: 0.70 ± 0.92 BOP: NR

* indicates *p* < 0.05; NR, not reported; FM, full mouth; PM, partial mouth; BL, baseline; PPD, probing pocket depth; CAL, clinical attachment level; BOP, bleeding on probing; SRP, scaling and root planing; U/S, ultrasonic scaling; CHX, chlorhexidine; DH, doxycycline hyclate; MINO, minocycline; aPDT, antimicrobial photodynamic therapy; MB, methylene blue; ICG, indocyanine green; PC, phenothiazine chloride; PVP-I, povidone-iodine; TBO, toluidine blue O.

**Table 3:** Clinical outcome assessment and adverse events of the included studies.

Study Citation	Blinding	Examiners	Calibration	Probing Type	Use of Stent	Gingival Bleeding Indices	Plaque Indices	Adverse Events
Carvalho et al., 2007 [48]	single	2	Yes	Manual	NR	NR	NR	None, 2 chips dislodged and replaced
Grisi et al., 2002 [46]	single	1	NR	Computer assisted	yes	Loesche 1979	Loe 1967	gingival pain, discomfort, local irritation and gingival oedema, gingival abscesses 3 sites
Kasaj et al., 2007 [49]	single	1	Yes	Manual	NR	NR	NR	gingival discomfort and gingival swelling
Mizrak et al., 2006 [47]	single	NR	NR	NR	Yes	Ainamo and Bay 1975	Loe 1967	NR
Bogren et al., 2008 [44]	single	>1	Yes	Manual	NR	NR	NR	None
Shalev 2019 [45]	double	>1	Yes	Manual	NR	NR	NR	NR
van Steenberghe et al., 1999 [43]	double	6	NR	Manual	Yes	Muhlemann 1977	Silness and Loe 1964	8 minor, 3 redness, 3 abscesses
Killeen et al., 2018 [42]	single	2	Yes	Manual	NR	NR	NR	None
Megally et al., 2020 [41]	single	3	Yes	Force controlled	NR	Muhlemann 1977	Lange et al., 1977	None
Müller Campanile et al., 2015 [50]	single	1	NR	NR	NR	NR	Silness and Loe 1964	2–pain/discomfort
Katsikanis et al., 2020 [51]	single	1	NR	NR	NR	Loe and Silness 1963	Silness and Loe 1964	None
Sukumar et al., 2020 [52]	single	1	Yes	Manual	NR	NR	O′Leary 1972	None
Lulic et al., 2009 [40]	single	1	Yes	Manual	Yes	NR	NR	NR
Petelin et al., 2015 [39]	double	1	NR	Force controlled	NR	NR	Silness and Loe 1964	NR
Harmouche et al., 2019 [53]	single	1	Yes	NR	NR	NR	NR	None
Kessler et al., 2021 [54]	single	NR	Yes	Force controlled	Yes	Ainamo and Bay 1975	O′Leary 1972	None

NR, not reported.

## Data Availability

The data presented in this study are available in Supplementary Material File S1.

## Supplementary Materials

The following are available online at https://www.mdpi.com/article/10.3390/antibiotics10101178/s1, File S1: Search strategies for each electronic database, Table S1: Studies excluded after full-text screening and reasons for exclusion, Table S2: Judgment of each risk of bias item for each included study according ROB2.
