# Supplementary material for: Clinical Efficacy of Repeated Applications of Local Drug Delivery and Adjunctive Agents in Nonsurgical Periodontal Therapy: A Systematic Review
Source: Antibiotics (Basel). 2021 Sep 28;10(10):1178. doi: 10.3390/antibiotics10101178 (PMC8532920; doi:10.3390/antibiotics10101178)
Supplement: Supplementary file 1 [file antibiotics-10-01178-s001.zip › antibiotics-1362420-supplementary.pdf]

Supplementay Materials

Clinical Efficacy of Repeated Applications of Local Drug Delivery and Adjunctive Agents in Nonsurgical Periodontal Therapy: A Systematic Review

Oi Leng Tan, Syarida Hasnur Safii and Masfueh Razali *

File S1: Search strategies for each electronic database.

PubMed (1979 to July 2021)

1. (((((periodontal diseases [mesh]) NOT periodontal cyst [mesh]) NOT gingival diseases [mesh]) NOT periapical diseases [mesh]) NOT peri-implantitis [mesh]) OR periodontitis [tw]
2. (dental prophylaxis [mesh] OR subgingival curettage [mesh] OR dental prophylaxis [tw] OR scaling root planing [tw] OR dental scaling [tw] OR root planing [tw] OR dental cleaning [tw] OR SRP [tw] OR scaling planing [tw] OR ultrasonic therapy [tw] OR ultrasonic therapy [mesh] OR ultrasonic scaling [tw] OR root surface debridement [tw] OR root surface instrumentation [tw] OR periodontal debridement [tw] OR nonsurgical [tw] OR non-surgical [tw]) NOT (ultrasonic surgical procedures [mesh])
3. (randomized controlled trial[pt] OR controlled clinical trial [pt] OR randomized [tiab] OR placebo [tiab] OR clinical trials as topic [mesh:noexp] OR randomly [tiab] OR trial[ti] NOT (animals [mh] NOT humans [mh])) NOT (review [pt] OR systematic review [pt]) [Adapted from Lefebvre et al. (2019)]
4. (adjunct* [tw]) AND (dental enamel proteins [mh] OR enamel matrix derivative* [tw] OR Anti-infective agents [mh] OR antibiotic* [tw] OR antiseptic* [tw] OR antimicrobial* [tw] OR antibacterial*[tw] OR tetracycline [tw] OR chlorhexidine [tw] OR doxycycline [tw] OR metronidazole [tw] OR minocycline [tw] OR hyaluron* [tw] OR probiotic* [tw] OR photodynamic therap* [tw])
5. 1 and 2 and 3 and 4

MEDLINE via OVID (1946 to July 2021)

1. exp Periodontal Diseases/ or periodonti$.mp. or (periodontal adj3 pocket$).mp. or "periodont$ attachment loss".mp.

2. exp Dental Prophylaxis/ or exp subgingival curettage/ or exp ultrasonic therapy/ or ((dental or oral or teeth or tooth or subgingival) adj6 (scaling or scale$ or curettage)).mp. or (dental prophylaxis or oral prophylaxis).mp. or (root adj (plane$ or planing)).mp. or (periodontal adj debridement$).mp. or (root surface adj debridement$).mp. or (root surface adj instrumentation$).mp. or (non?surgical adj3 periodontal).mp.
3. adjunct$.mp.
4. exp Anti-infective agents/ or (anti?biotic$ or anti?septic$ or anti?bacterial$ or anti?microbial$).mp. or (tetracycline or chlorhexidine or doxycycline or metronidazole or minocycline or hyaluron$ or photodynamic or enamel matrix derivative$ or probiotic$).mp.

6. randomized controlled trial.pt. or controlled clinical trial.pt. or randomized.ab. or placebo.ab. or drug therapy.fs. or randomly.ab. or trial.ab. or groups.ab. NOT (review.pt or systematic review.pt) [Adapted from Lefebvre et al. (2019)]
5. 1 and 2 and 3 and 4 and 5
6. (exp animal/ or animal.hw. or nonhuman/) not humans.sh.
7. 6 not 7

EMBASE via OVID (1974 to July 2021)

1. exp Periodontal Diseases/ or periodonti$.mp. or (periodontal adj3 pocket$).mp. or "periodont$ attachment loss".mp.
2. exp Dental Prophylaxis/ or exp subgingival curettage/ or exp ultrasonic therapy/ or ((dental or oral or teeth or tooth or subgingival) adj6 (scaling or scale$ or curettage)).mp. or (dental prophylaxis or oral prophylaxis).mp. or (root adj (plane$ or planing)).mp. or (periodontal adj debridement$).mp. or (root surface adj debridement$).mp. or (root surface adj instrumentation$).mp. or (non?surgical adj3 periodontal).mp.
3. adjunct$.mp.
4. exp Anti-infective agents/ or (anti?biotic$ or anti?septic$ or anti?bacterial$ or anti?microbial$).mp. or (tetracycline or chlorhexidine or doxycycline or metronidazole or minocycline or hyaluron$ or photodynamic or enamel matrix derivative$ or probiotic$).mp.
5. Randomized controlled trial/ or Controlled clinical study/ or random$.ti,ab. or randomisation/ or intermethod comparison/ or placebo.ti,ab. or (compare or compared or comparison).ti. or ((evaluated or evaluate or evaluating or assessed or assess) and (compare or compared or comparing or comparison)).ab. or (open adj label).ti,ab. or ((double or single

or doubly or singly) adj (blind or blinded or blindly)).ti,ab. or double blind procedure/ or parallel group$1.ti,ab. or (crossover or cross over).ti,ab. or ((assign$ or match or matched or allocation) adj5 (alternate or group$1 or intervention$1 or patient$1 or subject$1 or participant$1)).ti,ab. or (assigned or allocated).ti,ab. or (controlled adj7 (study or design or trial)).ti,ab. or (volunteer or volunteers).ti,ab. or human experiment/ or trial.ti. [Adapted from Lefebvre et al. (2019)]

6. random$ adj sampl$ adj7 (cross section$ or questionnaire$1 or survey$ or database$1).ti,ab. not (comparative study/ or controlled study/ or randomi?ed controlled.ti,ab. or randomly assigned.ti,ab.) or Cross-sectional study/ not (randomized controlled trial/ or controlled clinical study/ or controlled study/ or randomi?ed controlled.ti,ab. or control group$1.ti,ab.) or (((case adj control$) and random$) not randomi?ed controlled).ti,ab. or (Systematic review not (trial or study)).ti. or (nonrandom$ not random$).ti,ab. or (Random field$).ti,ab. or (random cluster adj3 sampl$).ti,ab. or (review.ab. and review.pt.) not trial.ti. or (we searched).ab. and (review.ti. or review.pt.) or (update review).ab. or (databases adj4 searched).ab. or (rat or rats or mouse or mice or swine or porcine or murine or sheep or lambs or pigs or piglets or rabbit or rabbits or cat or cats or dog or dogs or cattle or bovine or monkey or monkeys or trout or marmoset$1).ti. and animal experiment/ or Animal experiment/ not (human experiment/ or human/)
7. 5 not 6
8. 1 and 2 and 3 and 4 and 7

Cochrane Central Register of Controlled Clinical Trials (CENTRAL) (inception to July 2021)

1. MeSH descriptor: [Periodontal Diseases] explode all trees
2. (periodonti*) OR (periodontal near/3 pocket*) OR (periodont* attachment loss)
3. #1 or #2
4. MeSH descriptor: [Dental Prophylaxis] explode all trees
5. (dental or oral or teeth or tooth or subgingival) AND (scaling or scale* or curettage)
6. (dental prophylaxis OR oral prophylaxis) OR (root* NEXT plane* or root* NEXT planing) OR (periodontal NEXT debridement*)
7. (root surface NEXT debridement*) OR (root surface NEXT instrumentation*) OR ((non surgical or non-surgical or nonsurgical) near/3 periodontal)
8. #4 or #5 or #6 or #7
9. (adjunct*)

10. MeSH descriptor: [Anti-Infective Agents] explode all trees
11. (anti biotic* or anti septic* or anti bacterial* or anti microbial* or antibiotic* or antiseptic* or antibacterial* or antimicrobial* or anti-biotic* or anti-septic* or anti-bacterial* or anti-microbial*) OR (tetracycline or chlorhexidine or doxycycline or metronidazole or minocycline or hyaluron* or photodynamic or enamel matrix derivative* or probiotic*)
12. #10 or #11
13. #3 and #8 and #9 and #12

Web of Science (All databases) (1980 to July 2021)

1. TS=((periodonti*) OR (periodontal near/3 pocket*) OR (periodont* attachment loss))
2. TS=((dental or oral or teeth or tooth or subgingival) AND (scaling or scale* or curettage))
3. TS=((dental prophylaxis OR oral prophylaxis) OR (root* NEAR/1 plane* or root* NEAR/1 planing) OR (periodontal NEAR/1 debridement*))
4. TS=((root surface debridement*) OR (root surface instrumentation*) or (non surgical or non-surgical or nonsurgical))
5. #4 OR #3 OR #2
6. TS=(anti biotic* or anti septic* or anti bacterial* or anti microbial* or antibiotic* or antiseptic* or antibacterial* or antimicrobial* or anti-biotic* or anti-septic* or anti-bacterial* or anti-microbial*)
7. TS=(tetracycline or chlorhexidine or doxycycline or metronidazole or minocycline or hyaluron* or photodynamic or enamel matrix derivative* or probiotic*)
8. #7 OR #6
9. TS=(adjunct*)
10. TS=(randomized controlled trial or randomised controlled clinical trial OR random* OR placebo OR clinical trial*)
11. TI=(review)
12. #10 AND #9 AND #8 AND #5 AND #1
13. #12 NOT #11
14. TS=(animal* NOT human*)
15. #13 NOT #14

US National Institutes of Health Ongoing Trials Register (ClinicalTrials.gov) (inception to July 2021)

Advanced search:

Condition: periodontitis

Other terms: adjunct

Applied filter: Interventional

World Health Organization International Clinical Trials Registry Platform (inception to July 2021)

Periodontitis AND adjunct

ProQuest Dissertations & Theses Global (1879 to July 2021)

1. ((periodonti*) OR ("periodontal near/3 pocket*") OR ("periodont* attachment loss")) NOT ti(implant* or gingivitis or mucositis or orthodont* or endodont*)
2. ("dental prophylaxis" OR "oral prophylaxis") OR ("root* NEAR/1 plane*" OR "root* NEAR/1 planing") OR ("periodontal NEAR/1 debridement*") OR ("root surface debridement*") OR ("root surface instrumentation*") OR ("non surgical" or "non-surgical" or "nonsurgical") OR (dental or oral or teeth or tooth or subgingival) AND (scaling or scale* or curettage)
3. adjunct*
4. (anti?biotic* or anti?septic* or anti?bacterial* or anti?microbial*) OR (tetracycline or chlorhexidine or doxycycline or metronidazole or minocycline or hyaluron* or photodynamic or enamel matrix derivative* or probiotic*)
5. randomi?ed control* trial OR random* OR placebo OR clinical trial*
6. ti(review OR (in?vitro) OR (ex?vivo) OR (in?vivo) OR (cross?section*) OR retrospective OR observational OR experiment* OR literature)
7. s1 and s2 and s3 and s4 and s5
8. s7 not s6
9. su((animal*) NOT (human*))
10. s8 not s9

Table S1. Studies excluded after full-text screening and reasons for exclusion

No	Author(s)	Year	Title of study	Journal	Volume	Issue	Page	Exclusion reason(s)
1	Ahamed, S.; Jalaluddin, M.; Khalid, I.; Moon, N.; Shaf, T. K.; Ali, F. M.	2013	The use of controlled release locally delivered 10% doxycycline hyclate gel as an adjunct to scaling and root planing in the treatment of chronic periodontitis: clinical and microbiological results	Journal of Contemporary Dental Practice	14	6	1080-1086	No randomisation
2	Aimetti, M.; Romano, F.; Guzzi, N.; Carnevale, G.	2011	One-stage full-mouth disinfection as a therapeutic approach for generalized aggressive periodontitis	Journal of Periodontology	82	6	845-853	No control
3	Aimetti, M.; Romano, F.; Torta, I.; Cirillo, D.; Caposio, P.; Romagnoli, R.	2004	Debridement and local application of tetracycline-loaded fibres in the management of persistent periodontitis: Results after 12 months	Journal of Clinical Periodontology	31	3	166-172	Discontinued adjunct
4	Akıncıbay, H., Şenel, S., & Ay, Z. Y.	2007	Application of chitosan gel in the treatment of chronic periodontitis.	Journal of Biomedical Materials Research. Part B: Applied Biomaterials	80	2	290-296	Adjunct not commercially available
5	Al Habashneh, R., Mashal, M. A., Khader, Y., & Qudah, R.	2019	Clinical and biological effects of adjunctive photodynamic therapy in refractory periodontitis	Journal of Lasers in Medical Sciences	10	2	139-145	No randomisation

6	Balata, M. L., Andrade, L. P. D., Santos, D. B. N., Cavalcanti, A. N., Tunes, U. D. R., Ribeiro, E. D. P., & Bittencourt, S.	2013	Photodynamic therapy associated with full-mouth ultrasonic debridement in the treatment of severe chronic periodontitis: a randomized-controlled clinical trial	Journal of Applied Oral Science: Revista FOB	21	2	208-214	Failing sites retreated per initial basic therapy
7	Bizzarro, S., Van der Velden, U., & Loos, B. G.	2016	Local disinfection with sodium hypochlorite as adjunct to basic periodontal therapy: A randomized controlled trial	Journal of Clinical Periodontology	43	9	778-788	Used systemic antimicrobials
8	Blomlöf, L., Bergman, E., Forsgårdh, Å., Foss, L., Larsson, A., Sjöberg, B., Uhlander, L., et al.	2000	A clinical study of root surface conditioning with an EDTA gel. I. Nonsurgical periodontal treatment.	International Journal of Periodontics & Restorative Dentistry	20	6	561-565	Experimental adjunct
9	Bosco, J., Lopes, B., Bosco, A. F., Spolidorio, D. M., & Marcantonio, R. A.	2009	Local application of tetracycline solution with a microbrush: An alternative treatment for persistent periodontitis	Quintessence International	40	1	29-40	Adjunct not commercially available
10	Bundidpun, P., Srisuwantha, R., & Laosrisin, N.	2018	Clinical effects of photodynamic therapy as an adjunct to full-mouth ultrasonic scaling and root planing in treatment of chronic periodontitis	Laser Therapy	27	1	33-39	Adjuncts administered one week after SRP
11	Cappuyns, I., Cionca, N., Wick, P., Giannopoulou, C., & Mombelli, A.	2012	Treatment of residual pockets with photodynamic therapy, diode laser, or deep	Lasers in Medical Science	27	5	979-986	Not used as adjunct

			scaling. A randomized, split-mouth controlled clinical trial					
12	Carvalho, V.F., Andrade, P.V., Rodrigues, M.F., Hirata, M.H., Hirata, R.D., Pannuti, C.M., De Micheli, G. et al.	2015	Antimicrobial photodynamic effect to treat residual pockets in periodontal patients: a randomized controlled clinical trial	Journal of Clinical Periodontology	42	5	440-447	Not used as adjunct
13	Chiang, C. P., Hsieh, O., Tai, W. C., Chen, Y. J., & Chang, P. C.	2019	Clinical outcomes of adjunctive indocyanine green-diode lasers therapy for treating refractory periodontitis: A randomized controlled trial with in vitro assessment.	Journal of The Formosan Medical Association				Incomplete data
14	Cortelli, J.R., Aquino, D.R., Cortelli, S.C., Carvalho-F, J., Roman-Torres, C.V., & Costa, F.O.	2008	A double-blind randomized clinical trial of subgingival minocycline for chronic periodontitis.	Journal of Oral Science	50	3	259-265	No CAL data
15	Cortelli, J. R., Querido, S. M. R., Aquino, D. R., Ricardo, L. H., & Pallos, D.	2006	Longitudinal clinical evaluation of adjunct minocycline in the treatment of chronic periodontitis	Journal of Periodontology	77	2	161-166	No CAL data
16	Cortelli, S. C., Cavallini, F., Alves, M. F. R., Bezerra, A. A., Queiroz, C. S., & Cortelli, J. R.	2009	Clinical and microbiological effects of an essential-oil-containing mouth rinse applied in the “one-stage full-mouth disinfection” protocol—a randomized doubled-blinded preliminary study.	Clinical Oral Investigations	13	2	189-194	No CAL data

17	Cortelli, S.C., Cortelli, J.R., Holzhausen, M., Franco, G.C.N., Rebelo, R.Z., Sonagere, A.S., Queiroz, C.D.S. et al.	2009	Essential oils in one-stage full-mouth disinfection: double-blind, randomized clinical trial of long-term clinical, microbial and salivary effects.	Journal of Clinical Periodontology	36	4	333-342	No CAL data
18	Cosyn, Jan; Wyn, Iris; De Rouck, Tim; Sabzevar, Mehran Moradi	2006	Long-term clinical effects of a chlorhexidine varnish implemented treatment strategy for chronic periodontitis	Journal of Periodontology	77	3	406-415	No CAL data
19	da Cruz Andrade, P.V., Alves, V.T.E., de Carvalho, V.F., Rodrigues, M.D.F., Pannuti, C.M., Holzhausen, M., De Micheli, G. et al.	2017	Photodynamic therapy decrease immune-inflammatory mediators levels during periodontal maintenance	Lasers in Medical Science	32	1	9-17	Not used as adjunct
20	D'aiuto, F., Parkar, M., Nibali, L., Suvan, J., Lessem, J., & Tonetti, M. S.	2006	Periodontal infections cause changes in traditional and novel cardiovascular risk factors: Results from a randomized controlled clinical trial	American Heart Journal	151	5	977-984	No PPD/CAL data
21	Dannewitz, B., Lippert, K., Lang, N. P., Tonetti, M. S., & Eickholz, P.	2009	Supportive periodontal therapy of furcation sites: Non-surgical instrumentation with or without topical doxycycline	Journal of Clinical Periodontology	36	6	514-522	Subset of Tonetti 2012
22	De Lissovoy, G., Rentz, A. M., Dukes, E. M., Eaton, C.	1999	The cost-effectiveness of a new chlorhexidine delivery system in the treatment of adult periodontitis	Journal of the American Dental Association	130	6	855-862	Extension of Jeffcoat 1998

	A., Jeffcoat, M. K., Killoy, W. J., & Finkelman, R. D.							
23	Dean, J. W., Branch-Mays, G. L., Hart, T. C., Reinhardt, R. A., Shapiro, B., Santucci, E. A., & Lessem, J.	2003	Topically applied minocycline microspheres: why it works.	Compendium of Continuing Education in Dentistry	24	4	247-258	No control
24	Del Peloso Ribeiro, É., Bittencourt, S., Ambrosano, G. M. B., Nociti Jr, F. H., Sallum, E. A., Sallum, A. W., & Casati, M. Z.	2006	Povidone-iodine used as an adjunct to non-surgical treatment of furcation involvements.	Journal of Periodontology	77	2	211-217	Failing sites retreated per initial basic therapy
25	Drisko, C.L., Cobb, C.M., Killoy, W.J., Michalowicz, B.S., Pihlstrom, B.L., Lowenguth, R.A., Caton, J.G., et al.	1995	Evaluation of periodontal treatments using controlled-release tetracycline fibers: clinical response	Journal of Periodontology	66	8	692-699	Discontinued adjunct
26	DRKS00005389	2014	Clinical outcomes of adjunctive antimicrobial photodynamic therapy in the treatment of patients with severe chronic periodontitis - a controlled randomized clinical trial	http://www.who.int/trialsearch/trial2.aspx?trialid=drks00005389	Incomplete data			
27	Eickholz, P., Kim, T.S., Bürklin, T., Schacher, B.,	2002	Non-surgical periodontal therapy with adjunctive topical doxycycline: a double-blind randomized controlled multicenter study	Journal of Clinical Periodontology	29	2	108-117	15% DH not commercially available

	Renggli, H.H., Schaecken, M.T., Holle, R., et al.							
28	Feng, H.S., Bernardo, C.C., Sonoda, L.L., Hayashi, F., Romito, G.A., De Lima, L.A.P.A., Lotufo, R.F.M. et al.	2011	Subgingival ultrasonic instrumentation of residual pockets irrigated with essential oils: a randomized controlled trial.	Journal of Clinical Periodontology	38	7	637-643	Adjuncts administered one week after SRP
29	Flemmig, T. F., Weinacht, S., Rüdiger, S., Rumetsch, M., Jung, A., & Klaiber, B.	1996	Adjunctive controlled topical application of tetracycline HCl in the treatment of localized persistent or recurrent periodontitis: Effects on clinical parameters and elastase-α1-proteinase inhibitor in gingival crevicular fluid.	Journal of Clinical Periodontology	23	10	914-921	Discontinued adjunct
30	Friesen, L. R., Williams, K. B., Krause, L. S., & Killoy, W. J.	2002	Controlled local delivery of tetracycline with polymer strips in the treatment of periodontitis.	Journal of Periodontology	73	1	13-19	Adjunct not commercially available
31	Fulvi, L., Polizzi, E. & Calderini, A.	2011	Effectiveness of local antibiotic therapy plus scaling/root-planing: An experimental split-mouth study	Prevenzione E Assistenza Dentale	37	2	33-43	Adjunct not commercially available
32	Garrett, S., Johnson, L., Drisko, C.H., Adams, D.F., Bandt, C., Beiswanger, B., Bogle, G., et al.	1999	Two multi-center studies evaluating locally delivered doxycycline hyclate, placebo control, oral hygiene, and scaling and root planing in the treatment of periodontitis.	Journal of Periodontology	70	5	490-503	Not used as adjunct

33	Garrett, S., Adams, D.F., Bogle, G., Donly, K., Hastings Drisko, C., Hallmon, W.W., Brady Hancock, E., et al.	2000	The effect of locally delivered controlled-release doxycycline or scaling and root planing on periodontal maintenance patients over 9 months.	Journal of Periodontology	71	1	22-30	Subset of Garrett 1999
34	Giannelli, M., Formigli, L., Lorenzini, L., & Bani, D.	2012	Combined photoablative and photodynamic diode laser therapy as an adjunct to non-surgical periodontal treatment. A randomized split-mouth clinical trial	Journal of Clinical Periodontology	39	10	962-970	Adjunct administered one week after SRP
35	Giannelli, M., Formigli, L., Lorenzini, L., & Bani, D.	2015	Efficacy of combined photoablative-photodynamic diode laser therapy adjunctive to scaling and root planing in periodontitis: randomized split-mouth trial with 4-year follow-up	Photomedicine and Laser Surgery	33	9	473-480	Adjunct administered one week after SRP
36	Giannelli, M., Materassi, F., Fossi, T., Lorenzini, L., & Bani, D.	2018	Treatment of severe periodontitis with a laser and light-emitting diode (LED) procedure adjunctive to scaling and root planing: a double-blind, randomized, single-center, split-mouth clinical trial investigating its efficacy and patient-reported outcomes at 1 year.	Lasers in Medical Science	33	5	991-1002	Adjunct administered one week after SRP
37	Giannopoulou, C., Cappuyns, I., Cancela, J., Cionca, N., & Mombelli, A.	2012	Effect of photodynamic therapy, diode laser, and deep scaling on cytokine and acute-phase protein levels in gingival crevicular fluid of residual periodontal pockets	Journal of Periodontology	83	8	1018-1027	Not used as adjunct

38	Goh, E. X., Tan, K. S., Chan, Y. H., & Lim, L. P.	2017	Effects of root debridement and adjunctive photodynamic therapy in residual pockets of patients on supportive periodontal therapy: A randomized split-mouth trial	Photodiagnosis And Photodynamic Therapy	18		342-348	Failing sites retreated per initial basic therapy
39	Gonzales, J. R., Harnack, L., Schmitt-Corsitto, G., Boedeker, R. H., Chakraborty, T., Domann, E., & Meyle, J.	2011	A novel approach to the use of subgingival controlled-release chlorhexidine delivery in chronic periodontitis: A randomized clinical trial	Journal of Periodontology	82	8	1131-1139	Adjunct administered before SRP
40	Gopinath, V., Ramakrishnan, T., Emmadi, P., Ambalavanan, N., & Mammen, B.	2009	Effect of a controlled release device containing minocycline microspheres on the treatment of chronic periodontitis: A comparative study	Journal of Indian Society of Periodontology	13	2	79	No mention of method of randomisation
41	Grzech-Leśniak, K., Gaspirc, B., & Sculean, A.	2019	Clinical and microbiological effects of multiple applications of antibacterial photodynamic therapy in periodontal maintenance patients. A randomized controlled clinical study.	Photodiagnosis And Photodynamic Therapy	27		44-50	No CAL data
42	Hagiwara, S., Iida, M., & Ishikawa, I.	1993	Clinical and microbiological study concerning effects of scaling and root planing. 2. The effects of mechanical debridement until one year and of minocycline application topically.	Kokubyo Gakkai Zasshi. The Journal of The Stomatological Society, Japan	60	2	301-312	No mention of method of randomisation

43	Henke, C.J.; Genco, R.J.; Killoy, W.J.; Miller, D.P.; Evans, C.J.; Finkelman, R.D.; Villa, K.F. et al.	2001	An economic evaluation of a chlorhexidine chip for treating chronic periodontitis: the CHIP (chlorhexidine in periodontitis) study	Journal of The American Dental Association (1939)	132	11	1557-1569	No final PPD/CAL reported
44	Ioannou, I., Dimitriadis, N., Papadimitriou, K., Vouros, I., Sakellari, D., & Konstantinidis, A.	2011	The effect of locally delivered doxycycline in the treatment of chronic periodontitis. A clinical and microbiological cohort study.	Journal of Oral & Maxillofacial Research	1	4	e1	No randomisation, no control
45	Jentsch, H.R., Roccuzzo, M., Marini, L., Kasaj, A., Fimmers, R., Jepsen, S.	2018	0059: Flapless application of enamel matrix derivative (EMD) as an adjunct to scaling and root planing – a multicenter RCT	Journal of Clinical Periodontology	45		26-27	Less than 6 months duration
46	Jeffcoat, M. K., Palcanis, K. G., Weatherford, T. W., Reese, M., Geurs, N. C., & Flashner, M.	2000	Use of a biodegradable chlorhexidine chip in the treatment of adult periodontitis: clinical and radiographic findings.	Journal of Periodontology	71	2	256-262	Subset of Jeffcoat 1998
47	Jeffcoat, M.K., Bray, K.S., Ciancio, S.G., Dentino, A.R., Fine, D.H., Gordon, J.M., Gunsolley, J.C., et al.	1998	Adjunctive use of a subgingival controlled-release chlorhexidine chip reduces probing depth and improves attachment level compared with scaling and root planing alone	Journal of Periodontology	69	9	989-997	Only adjunct repeated, not SRP; possible bias towards adjunct
48	Jones, A. A., Kornman, K. S., Newbold, D. A., & Manwell, M. A.	1994	Clinical and microbiological effects of controlled-release locally delivered minocycline in periodontitis	Journal of Periodontology	65	11	1058-1066	Adjunct not commercially available

49	Kanoriya, D., Singhal, S., Garg, V., Pradeep, A. R., Garg, S., & Kumar, A.	2018	Clinical efficacy of subgingivally-delivered 0.75% boric acid gel as an adjunct to mechanotherapy in chronic periodontitis: A randomized, controlled clinical trial.	Journal of Investigative and Clinical Dentistry	9	e1227 1		Adjunct not commercially available
50	Kerry, G.	1994	Tetracycline-loaded fibers as adjunctive treatment in periodontal disease.	Journal of The American Dental Association (1939)	125	9	1199-1203	Discontinued adjunct
51	Killeen, A. C., Harn, J. A., Erickson, L. M., Yu, F., & Reinhardt, R. A.	2016	Local minocycline effect on inflammation and clinical attachment during periodontal maintenance: Randomized clinical trial	Journal of Periodontology	87	10	1149-1157	Original study of Killeen 2018
52	Killoy, W. J.	1999	Assessing the effectiveness of locally delivered chlorhexidine in the treatment of periodontitis	Journal of The American Dental Association (1939)	130	4	567-570	Review
53	Kim, T. S., Burklin, T., Schacher, B., Ratka-Kruger, P., Schaecken, M. T., Renggli, H. H., & Eickholz, P.	2003	Local application of antibiotics versus mechanical debridement in supportive periodontal treatment.	Deutsche Zahnarztliche Zeitschrift	58	10	579-583	Not used as adjunct
54	Kinane, D. F., & Radvar, M.	1999	A six-month comparison of three periodontal local antimicrobial therapies in persistent periodontal pockets	Journal of Periodontology	70	1	1-7	Discontinued adjunct
55	Kolbe, M.F., Ribeiro, F.V., Luchesi, V.H., Casarin,	2014	Photodynamic therapy during supportive periodontal care: clinical, microbiologic,	Journal of Periodontology	85	8	e277-e286	Not used as adjunct

	R.C., Sallum, E.A., Nociti Jr, F.H., Ambrosano, G.M., et al.		immunoinflammatory, and patient-centered performance in a split-mouth randomized clinical trial					
56	Kondreddy, K., Ambalavanan, N., Ramakrishna, T., & Kumar, R. S.	2012	Effectiveness of a controlled release chlorhexidine chip (PerioColTM-CG) as an adjunctive to scaling and root planing when compared to scaling and root planing alone in the treatment of chronic periodontitis: A comparative study.	Journal of Indian Society of Periodontology	16	4	553-557	No randomisation
57	Konuganti, K.; Kumar, A.	2016	Efficacy of subgingivally delivered flurbiprofen and chlorhexidine chip in the treatment of chronic periodontitis – A randomized controlled clinical trial	International Journal of Pharmaceutical Sciences Review and Research	40	1	149-153	Adjunct not commercially available
58	Koshy, G., Kawashima, Y., Kiji, M., Nitta, H., Umeda, M., Nagasawa, T., & Ishikawa, I.	2005	Effects of single-visit full-mouth ultrasonic debridement versus quadrant-wise ultrasonic debridement	Journal of Clinical Periodontology	32	7	734-743	No passive control
59	Laleman, I., Pauwels, M., Quirynen, M., & Teughels, W.	2020	A dual-strain Lactobacilli reuteri probiotic improves the treatment of residual pockets: A randomized controlled clinical trial.	Journal of Clinical Periodontology	47		43-53	Administered use of lozenges at home
60	Lauenstein, M., Kaufmann, M., & Persson, G. R.	2013	Clinical and microbiological results following nonsurgical periodontal therapy with or	Clinical Oral Investigations	17	7	1645-1660	No CAL data

			without local administration of piperacillin/tazobactam					
61	Leonhardt, Å., Bergström, C., Krok, L., & Cardaropoli, G.	2006	Healing following ultrasonic debridement and PVP-iodine in individuals with severe chronic periodontal disease: a randomized, controlled clinical study.	Acta Odontologica Scandinavica	64	5	262-266	PD in %
62	Leonhardt, Å., Bergström, C., Krok, L., & Cardaropoli, G.	2007	Microbiological effect of the use of an ultrasonic device and iodine irrigation in patients with severe chronic periodontal disease: a randomized controlled clinical study.	Acta Odontologica Scandinavica	65	1	52-59	No PPD/CAL data
63	Lie, T., Bruun, G., & Böe, O. E.	1998	Effects of topical metronidazole and tetracycline in treatment of adult periodontitis.	Journal of Periodontology	69	7	819-827	Discontinued adjunct
64	Machion, L., Andia, D. C., Lecio, G., Nociti Jr, F. H., Casati, M. Z., Sallum, A. W., & Sallum, E. A.	2006	Locally delivered doxycycline as an adjunctive therapy to scaling and root planing in the treatment of smokers: A 2-year follow-up	Journal of Periodontology	77	4	606-613	Extension of Machion 2004
65	McColl, E., Patel, K., Dahlen, G., Tonetti, M., Graziani, F., Suvan, J., & Laurell, L.	2006	Supportive periodontal therapy using mechanical instrumentation or 2% minocycline gel: a 12 month randomized, controlled, single masked pilot study.	Journal of Clinical Periodontology	33	2	141-150	Not used as adjunct
66	Meinberg, T. A., Barnes, C. M., Dunning, D. G., & Reinhardt, R. A.	2002	Comparison of conventional periodontal maintenance versus scaling and root planing with subgingival minocycline.	Journal of Periodontology	73	2	167-172	No randomisation

67	Modanese, D. D. G., Tiosso-Tamburi, R., de Goes, V. F. F., de Cássia Bergamaschi, C., Martinez, E. F., Napimoga, M. H., et al.	2016	Clinical and immunoinflammatory evaluation of one-stage full-mouth ultrasonic debridement as a therapeutic approach for smokers with generalized aggressive periodontitis: A short-term follow-up study	Journal of Periodontology	87	9	1012-1021	Not used as adjunct
68	Mongardini, C., van Steenberghe, D., Dekeyser, C., & Quirynen, M.	1999	One stage full- versus partial-mouth disinfection in the treatment of chronic adult or generalized early-onset periodontitis. I. Long-term clinical observations.	Journal of Periodontology	70	6	632-645	Comparison of different SRP
69	Müller, N., Moëne, R., Cancela, J. A., & Mombelli, A.	2014	Subgingival air-polishing with erythritol during periodontal maintenance: randomized clinical trial of twelve months.	Journal of Clinical Periodontology	41	9	883-889	CHX content not with the intention to have a therapeutic effect
70	Muzaheed, M., Acharya, S., Hakami, A.R., Allemailem, K.S., Alqahtani, K., Al Saffan, A., Aldakheel, F.M. and Divakar, D.D.	2020	Effectiveness of single versus multiple sessions of photodynamic therapy as adjunct to scaling and root planing on periodontopathogenic bacteria in patients with periodontitis	Photodiagnosis and photodynamic therapy	32		102035	No passive control
71	NCT02487186	2015	Locally delivered doxycycline adjunct to nonsurgical periodontal therapy	https://clinicaltrials.gov/show/nct02487186	Incomplete data			

72	NCT04036890	2019	Local minocycline in patients under supportive periodontal therapy	https://clinicaltrials.gov/ct2/show/nct04036890	Incomplete data			
73	Newman, M. G., Kornman, K. S., & Doherty, F. M.	1994	A 6-month multi-center evaluation of adjunctive tetracycline fiber therapy used in conjunction with scaling and root planing in maintenance patients: Clinical results	Journal of Periodontology	65	7	685-691	Discontinued adjunct
74	Okuda, K., Wolff, L., Oliver, R., Osbom, J., Stoltenberg, J., Bereuter, J., Anderson, L., et al.	1992	Minocycline slow-release formulation effect on subgingival bacteria.	Journal of Periodontology	63	2	73-79	No PPD/CAL data
75	Oringer, R. J., Van, T. D., & Lessem, J.	2002	The challenge of treating periodontal patients who smoke - the efficacy of Arestin	Journal of The International Academy of Periodontology	4	3	89-94	Mix subpopulation of 2 RCTs
76	Paquette, D., Oringer, R., Lessem, J., Offenbacher, S., Genco, R., Persson, G.R., Santucci, E.A., et al.	2003	Locally delivered minocycline microspheres for the treatment of periodontitis in smokers.	Journal of Clinical Periodontology	30	9	787-794	Subset of Williams 2001; no CAL data
77	Pedrazzoli, V., Kilian, M., & Karring, T.	1992	Comparative clinical and microbiological effects of topical subgingival application of metronidazole 25% dental gel and scaling in the treatment of adult periodontitis	Journal of Clinical Periodontology	19	9	715-722	Not used as adjunct

78	Pejčić, A., Kojović, D., Minić, I., Mirković, D., Denić, M., & Stojanović, M.	2015	Therapeutic efficacy of clindamycin gel as an adjunct to scaling and root planing therapy in chronic periodontal disease	Acta Clinica Croatica	54	1	46-50	Adjunct not commercially available
79	Pietruska, M., Paniczko, A., Waszkiel, D., Pietruski, J., & Bernaczyk, A.	2006	Efficacy of local treatment with chlorhexidine gluconate drugs on the clinical status of periodontium in chronic periodontitis patients	Advances in Medical Sciences	51	7	162-165	Adjunct administered one week after SRP
80	Quirynen, M., De Soete, M., Boschmans, G., Pauwels, M., Coucke, W., Teughels, W., & Van Steenberghe, D.	2006	Benefit of "one-stage full-mouth disinfection" is explained by disinfection and root planing within 24 hours: A randomized controlled trial	Journal of Clinical Periodontology	33	9	639-647	Comparison of supragingival antiseptics
81	Quirynen, M., Mongardini, C., De Soete, M., Pauwels, M., Coucke, W., Van Eldere, J., & Van Steenberghe, D.	2000	The role of chlorhexidine in the one-stage full-mouth disinfection treatment of patients with advanced adult periodontitis. Long-term clinical and microbiological observations.	Journal of Clinical Periodontology	27	8	578-589	Comparison of different SRP
82	Ratka-Krüger, P., Schacher, B., Bürklin, T., Böddinghaus, B., Holle, R., Renggli, H.H., Eickholz, P., et al.	2005	Non-surgical periodontal therapy with adjunctive topical doxycycline: a double-masked, randomized, controlled multicenter study. II. Microbiological results.	Journal of Periodontology	76	1	66-74	No PPD/CAL data

83	Raut, C. P., Sethi, K. S., Kohale, B. R., Mamajiwala, A., & Warang, A.	2018	Indocyanine green-mediated photothermal therapy in treatment of chronic periodontitis: A clinico-microbiological study.	Journal of Indian Society of Periodontology	22	3	221-227	Adjunct not commercially available
84	Renvert, S., Dahlén, G., & Snyder, B.	1997	Clinical and microbiological effects of subgingival antimicrobial irrigation with citric acid as evaluated by an enzyme immunoassay and culture analysis.	Journal of Periodontology	68	4	346-352	No randomisation
85	Renvert, S., Dahlen, G., & Wikström, M.	1996	Treatment of periodontal disease based on microbiological diagnosis. Relation between microbiological and clinical parameters during 5 years	Journal of Periodontology	67	6	562-571	Use of systemic antimicrobials
86	Ribeiro, É. D. P., Bittencourt, S., Sallum, E. A., Sallum, A. W., & Nociti Júnior, F. H.	2010	Non-surgical instrumentation associated with povidone-iodine in the treatment of interproximal furcation involvements	Journal of Applied Oral Science	18	6	599-606	Failing sites retreated per initial basic therapy
87	Rodrigues, I. F. G., Machion, L., Casati, M. Z., Nociti Jr, F. H., de Toledo, S., Sallum, A. W., & Sallum, E. A.	2007	Clinical evaluation of the use of locally delivered chlorhexidine in periodontal maintenance therapy	Journal of Periodontology	78	4	624-628	Adjunct administered 3 months after SRP
88	Romano, F., Torta, I., Debernardi, C., & Aimetti, M.	2005	Debridement and local application of tetracycline in the management of persistent	Minerva Stomatologica	54	1-2	43-51	Discontinued adjunct

			periodontitis. Clinical and microbiological results after 12 months.					
89	Rosling, B. G., Slots, J., Christersson, L. A., Gröndahl, H. G., & Genco, R. J.	1986	Topical antimicrobial therapy and diagnosis of subgingival bacteria in the management of inflammatory periodontal disease	Journal of Clinical Periodontology	13	10	975-981	Trial series
90	Rosling, B., Hellström, M. K., Ramberg, P., Socransky, S. S., & Lindhe, J.	2001	The use of PVP-iodine as an adjunct to non-surgical treatment of chronic periodontitis.	Journal of Clinical Periodontology	28	11	1023-1031	Failing sites retreated per initial basic therapy
91	Rudhart, A., Purucker, P., Kage, A., Hopfenmüller, W., & Bernimoulin, J. P.	1998	Local metronidazole application in maintenance patients. Clinical and microbiological evaluation	Journal of Periodontology	69	10	1148-1154	Not used as adjunct
92	Sakellari, D., Vouros, I., & Konstantinidis, A.	2003	The use of tetracycline fibres in the treatment of generalised aggressive periodontitis: clinical and microbiological findings	Journal of The International Academy of Periodontology	5	2	52-60	Discontinued adjunct
93	Santuchi, C. C., Cortelli, J. R., Cortelli, S. C., Cota, L. O. M., Fonseca, D. C., Alencar, C. O., & Costa, F. O.	2016	Scaling and root planing per quadrant versus one-stage full-mouth disinfection: assessment of the impact of chronic periodontitis treatment on quality of life - a clinical randomized, controlled trial	Journal of Periodontology	87	2	114-123	Comparison of different SRP

94	Santuchi, C. C., Cortelli, S. C., Cortelli, J. R., Cota, L. O. M., Alencar, C. O., & Costa, F. O.	2015	Pre- and post-treatment experiences of fear, anxiety, and pain among chronic periodontitis patients treated by scaling and root planing per quadrant versus one-stage full-mouth disinfection: A 6-month randomized controlled clinical trial	Journal of Clinical Periodontology	42	11	1024-1031	Comparison of different SRP
95	Schallhorn, R. A., cClain, P. K., Benhamou, V., Doobrow, J. H., Grandin, H. M., & Kasaj, A.	2020	Application of enamel matrix derivative in conjunction with non-surgical therapy for treatment of moderate to severe periodontitis: A twelve-month, randomized prospective, multi-center study	Journal of Periodontology				Inclusion of medically compromised patients
96	Sculean, A., Windisch, P., Keglevich, T., & Gera, I.	2003	Histologic evaluation of human intrabony defects following non-surgical periodontal therapy with and without application of an enamel matrix protein derivative.	Journal of Periodontology	74	2	153-160	Not RCT
97	Segarra-Vidal, M., Guerra-Ojeda, S., Vallés, L.S., López-Roldán, A., Mauricio, M.D., Aldasoro, M., Alpiste-Illueca, F. et al.	2017	Effects of photodynamic therapy in periodontal treatment: A randomized, controlled clinical trial.	Journal of Clinical Periodontology	44	9	915-925	Adjunct administered one week after SRP
98	Shaddox, L. M., Andia, D. C., Casati, M. Z., Nociti, F. H., Sallum, E. A.,	2007	Microbiologic changes following administration of locally delivered doxycycline in smokers: a 15-month follow-up.	Journal of Periodontology	78	11	2143-2149	Subset of Machion 2004, 2006

	Gollwitzer, J., & Walker, C. B.							
99	Shiloah, J., & Patters, M. R.	1996	Repopulation of periodontal pockets by microbial pathogens in the absence of supportive therapy	Journal of Periodontology	67	2	130-139	CAL data not separated by groups
100	Singh, M., Shreehari, A. K., Garg, P. K., & Singh, S.	2014	Clinical efficacy of chlorhexidine chips and tetracycline fibers as an adjunct to non surgical periodontal therapy	European Journal of General Dentistry	3	2	134-139	Adjunct administered one week after SRP
101	Soeroso, Y., Akase, T., Sunarto, H., Kemal, Y., Salim, R., Octavia, M., Viandita, A., et al.	2017	The risk reduction of recurrent periodontal pathogens of local application minocycline HCl 2% gel, used as an adjunct to scaling and root planing for chronic periodontitis treatment	Therapeutics and Clinical Risk Management	13		307-314	Duplicate of Soeroso 2016 which is more complete
102	Soskolne, W. A., Heasman, P. A., Stabholz, A., Smart, G. J., Palmer, M., Flashner, M., & Newman, H. N.	1997	Sustained local delivery of chlorhexidine in the treatment of periodontitis: A multi-center study.	Journal of Periodontology	68	1	32-38	Failing sites of control not retreated, bias towards adjunct?
103	Soskolne, W. A., Proskin, H. M., & Stabholz, A.	2003	Probing depth changes following 2 years of periodontal maintenance therapy including adjunctive controlled release of chlorhexidine.	Journal of Periodontology	74	4	420-427	No randomisation
104	Stelzel, M.	1997	Topical metronidazole application in recall patients: Long-term results	Journal of Clinical Periodontology	24	12	914-919	Not used as adjunct

105	Sweatha, C., Srikanth, C., & Babu, M. R.	2015	A comparative study of the effect of minocycline microspheres as an adjunct to scaling and root planing versus scaling and root planing alone in the treatment of chronic periodontitis	International Journal of Recent Scientific Research	6	4	3540-3550	Failing sites of control not retreated, bias towards adjunct?
106	Swierkot, K., Nonnenmacher, C. I., Mutters, R., Flores-de-Jacoby, L., & Mengel, R.	2009	One-stage full-mouth disinfection versus quadrant and full-mouth root planing	Journal of Clinical Periodontology	36	3	240-249	Comparison of different SRP
107	Timmerman, M. F., Van der Weijden, G. A., Van Steenbergen, T. J. M., Mantel, M. S., De Graaff, J., & Van der Velden, U.	1996	Evaluation of the long-term efficacy and safety of locally-applied minocycline in adult periodontitis patients	Journal of Clinical Periodontology	23	8	707-716	Subset of van Steenberghe 1999
108	Trojahn, M. R. B., Silva, R. C. D., & Joly, J. C.	2013	Non-surgical periodontal therapy for the treatment of chronic periodontitis	Rgo. Revista Gaúcha De Odontologia (Online)	61	4	529-534	Adjunct not commercially available
109	Tomasi, C., & Wennström, J. L.	2011	Locally delivered doxycycline as an adjunct to mechanical debridement at retreatment of periodontal pockets: outcome at furcation sites	Journal of Periodontology	82	2	210-218	Subset of Tomasi 2008
110	Tonetti, M. S., Cortellini, P., Carnevale, G., Cattabriga,	1998	A controlled multicenter study of adjunctive use of tetracycline periodontal fibers in	Journal of Clinical Periodontology	25	9	728-736	Discontinued adjunct

	M., De Sanctis, M., & Prato, G. P.		mandibular class II furcations with persistent bleeding					
111	Tonetti, M.S., Lang, N.P., Cortellini, P., Suvan, J.E., Eickholz, P., Fourmousis, I., Topoll, H., et al.	2012	Effects of a single topical doxycycline administration adjunctive to mechanical debridement in patients with persistent/recurrent periodontitis but acceptable oral hygiene during supportive periodontal therapy	Journal of Clinical Periodontology	39	5	475-482	Failing sites retreated per initial basic therapy
112	Ueda, M., Teranishi, Y., Nakagaki, N., Imai, H., Katagiri, E. & Funaoka, K.	1995	Periodontal therapy by local delivery of antibiotics - Periocline (R) administration in periodontal pocket in combination with scaling - (Part 4).	Oral Therapeutics and Pharmacology	14	2	99-105	Duration less than 6 months
113	Ueda, M., Ogata, C., Suga, H., Kohno, T., Imai, H., Cao, C., Geng, S., et al.	2001	Periodontal therapy with local delivery of antibiotics: Part 1 Observation at Osaka Dental University	Shikaigaku	64	2	187-190	Duration less than 6 months
114	Ueda, M., Ogata, C., Suga, H., Kohno, T., Imai, H., Cao, C., Geng, S., et al.	2001	Periodontal therapy with local delivery of antibiotics: Part 2 Observation at Beijing University	Shikaigaku	64	2	191-194	Duration less than 6 months
115	Unsal, E., Akkaya, M., & Walsh, T. F.	1994	Influence of a single application of subgingival chlorhexidine gel or tetracycline paste on the clinical parameters of adult periodontitis patients.	Journal of Clinical Periodontology	21	5	351-355	Duration less than 6 months

116	Vandekerckhove, B. N., Bollen, C. M., Dekeyser, C., Darius, P., & Quirynen, M.	1996	Full- versus partial-mouth disinfection in the treatment of periodontal infections. Long-term clinical observations of a pilot study	Journal of Periodontology	67	12	1251-1259	Comparison of different SRP
117	Vandekerckhove, B. N., Quirynen, M., & van Steenberghe, D.	1997	The use of tetracycline-containing controlled-release fibers in the treatment of refractory periodontitis.	Journal of Periodontology	68	4	353-361	Discontinued adjunct
118	Vitt, A., Gustafsson, A., Ramberg, P., Slizen, V., Kazeko, L. A., & Buhlin, K.	2019	Polyhexamethylene guanidine phosphate irrigation as an adjunctive to scaling and root planing in the treatment of chronic periodontitis.	Acta Odontologica Scandinavica	77	4	290-295	No CAL data
119	Wennström, J. L., Heijl, L., Dahlén, G., & Gröndahl, K.	1987	Periodic subgingival antimicrobial irrigation of periodontal pockets (I). Clinical observations.	Journal of Clinical Periodontology	14	9	541-550	Adjunct placed before SRP
120	Wong, M. Y., Lu, C. L., Liu, C. M., & Hou, L. T.	1999	Microbiological response of localized sites with recurrent periodontitis in maintenance patients treated with tetracycline fibers.	Journal of Periodontology	70	8	861-868	Discontinued adjunct
121	Wong, M. Y., Lu, C. L., Liu, C. M., Hou, L. T., & Chang, W. K.	1998	Clinical response of localized recurrent periodontitis treated with scaling, root planing, and tetracycline fiber.	Journal of The Formosan Medical Association = Taiwan Yi Zhi	97	7	490-497	Discontinued adjunct
122	Zingale, J., Harpenau, L., Bruce, G., Chambers, D., & Lundergan, W.	2012	The effectiveness of scaling and root planing with adjunctive time-release minocycline using an open and closed approach for the treatment of periodontitis.	General Dentistry	60	4	300-305	No CAL data, compared surgical procedure

123	Kumar, V., Singhal, R., Rastogi, P., Lal, N., Pandey, S., & Mahdi, A. A.	2021	Localized probiotic-guided pocket recolonization in the treatment of chronic periodontitis: a randomized controlled clinical trial	Journal of Periodontal & Implant Science	51	3	199	Duration less than 6 months
**Single application of LDA**								
123	Aboelsaad, N., Ghandour, R. & Abiad, R.	2014	Clinical efficacy of local delivered minocycline in the treatment of chronic periodontitis smoker patients.	Journal of Dentistry & Oral Health	1		1-5	
124	Ağan, S., Sönmez, S. & Serdar, M.	2006	The effect of topical doxycycline usage on gingival crevicular fluid MMP-8 levels of chronic and aggressive periodontitis patients: A pilot study	International Journal of Dental Hygiene	4	3	114-121	
125	Alwaeli, H. A., Al-Khateeb, S. N. & Al-Sadi, A.	2015	Long-term clinical effect of adjunctive antimicrobial photodynamic therapy in periodontal treatment: A randomized clinical trial	Lasers in Medical Science	30	2	801-807	
126	Azmak, N., Atilla, G., Luoto, H. & Sorsa, T.	2002	The effect of subgingival controlled-release delivery of chlorhexidine chip on clinical parameters and matrix metalloproteinase-8 levels in gingival crevicular fluid.	Journal of Periodontology	73	6	608-615	
127	Berakdar, M., Callaway, A., Fakhr Eddin, M., Roß, A. & Willershausen, B.	2012	Comparison between scaling-root-planing (SRP) and SRP/photodynamic therapy: Six-month study	Head and Face Medicine	8	1	2-7	

128	Betsy, J., Prasanth, C. S., Baiju, K. V., Presanthila, J. & Subhash, N.	2014	Efficacy of antimicrobial photodynamic therapy in the management of chronic periodontitis: A randomized controlled clinical trial	Journal of Clinical Periodontology	41		573–581	
129	Betsy, J., Prasanth, C. S., Baiju, K. V., Presanthila, J. & Subhash, N.	2016	Patients' perceptions of antimicrobial photodynamic therapy in the management of chronic periodontitis	Photodiagnosis Photodynamic Therapy	14		84-90	Related to Betsy 2014
130	Buduneli, E., Tünger, A., Evrenosoglu, E. & Bilgiç, A.	2001	Comparative clinical and microbiological effects of subgingival metronidazole application in adult periodontitis; 12-months results.	Journal of the International Academy of Periodontology	3	4	81-86	
131	Chondros, P., Nikolidakis, D., Christodoulides, N., Rössler, R., Gutknecht, N. & Sculean, A.	2009	Photodynamic therapy as adjunct to non-surgical periodontal treatment in patients on periodontal maintenance: A randomized controlled clinical trial.	Lasers in Medical Science	24	5	681–688	
132	Christodoulides, N., Nikolidakis, D., Chondros, P., Becker, J., Schwarz, F., Rössler, R., Sculean, A., et al.	2008	Photodynamic therapy as an adjunct to non-surgical periodontal treatment: A randomized, controlled clinical trial.	Journal of Periodontology	79	9	1638–1644	
133	Cosyn, J., Wyn, I., De Rouck, T. & Sabzevar, M. M.	2006	Long-term clinical effects of a chlorhexidine varnish implemented treatment strategy for chronic periodontitis	Journal of Periodontology	77	3	406–415	

134	Denez, E. M., Toma, S., Lasserre, J. F. & Brecx, M. C.	2016	Evaluation of a unique subgingival irrigation with 10% povidone-iodine after scaling and root planing: A randomized clinical trial.	Quintessence international (Berlin, Germany: 1985)	47	7	549–558	
135	Deo, V., Ansari, S., Mandia, S. & Bhongade, M.	2011	Therapeutic efficacy of subgingivally delivered doxycycline hyclate as an adjunct to non-surgical treatment of chronic periodontitis.	Journal of Oral and Maxillofacial Research	2	1	E3	
136	Derikvand, N., Ghasemi, S. S., Safiaghdam, H., Piriaei, H., & Chiniforush, N.	2020	Antimicrobial Photodynamic Therapy with Diode laser and Methylene blue as an adjunct to scaling and root planning: A clinical trial.	Photodiagnosis and photodynamic therapy	31		101818	
137	Dilsiz, A., Canakci, V. & Aydin, T.	2013	Clinical effects of potassium–titanyl–phosphate laser and photodynamic therapy on outcomes of treatment of chronic periodontitis: A randomized controlled clinical trial.	Journal of Periodontology	84	3	278–286	
138	do Vale, H. F., Casarin, R. C. V., Taiete, T., Bovi Ambrosano, G. M., Ruiz, K. G. S., Nociti, F. H., Sallum, E. A., et al.	2016	Full-mouth ultrasonic debridement associated with povidone iodine rinsing in GAgP treatment: a randomised clinical trial.	Clinical Oral Investigations	20	1	141–150	
139	Eick, S., Renatus, A., Heinicke, M., Pfister, W., Stratul, S.-I. & Jentsch, H.	2013	Hyaluronic acid as an adjunct after scaling and root planing: A prospective randomized clinical trial.	Journal of Periodontology	84	7	941–949	
140	Gandhi, K., Pavaskar, R., Cappetta, E. & Drew, H.	2019	Effectiveness of adjunctive use of low-level laser therapy and photodynamic therapy after	The International Journal of	39	6	837–843	

			scaling and root planing in patients with chronic periodontitis.	Periodontics & Restorative Dentistry				
141	Griffiths, G. S., Smart, G. J., Bulman, J. S., Weiss, G., Shrowder, J. & Newman, H. N.	2000	Comparison of clinical outcomes following treatment of chronic adult periodontitis with subgingival scaling or subgingival scaling plus metronidazole gel.	Journal of Clinical Periodontology	27	12	910–917	
142	Heasman, P. A., Heasman, L., Stacey, F. & McCracken, G. I.	2001	Local delivery of chlorhexidine gluconate (PerioChip) in periodontal maintenance patients.	Journal of Clinical Periodontology	28	1	90–95	
143	Henderson, R. J., Boyens, J. V., Holborow, D. W. & Pack, A. R. C.	2002	Scaling and root-planing treatment with adjunctive subgingival minocycline. A clinical pilot study over six months, of sites adjacent to and remote from the antibiotic application.	Journal of the International Academy of Periodontology	4	3	77–87	
144	Hill, G., Dehn, C., Hinze, A. V., Frentzen, M. & Meister, J.	2019	Indocyanine green-based adjunctive antimicrobial photodynamic therapy for treating chronic periodontitis: A randomized clinical trial.	Photodiagnosis and Photodynamic Therapy	26		29–35	
145	Isola, G., Matarese, G., Williams, R. C., Siciliano, V. I., Alibrandi, A., Cordasco, G. & Ramaglia, L.	2018	The effects of a desiccant agent in the treatment of chronic periodontitis: a randomized, controlled clinical trial.	Clinical Oral Investigations	22	2	791–800	

146	Jain, M., Dave, D., Jain, P., Manohar, B., Yadav, B. & Shetty, N.	2013	Efficacy of xanthan based chlorhexidine gel as an adjunct to scaling and root planing in treatment of the chronic periodontitis.	Journal of Indian Society of Periodontology	17	4	439–43	
147	Jain, R., Mohamed, F. & Hemalatha, M.	2012	Minocycline containing local drug delivery system in the management of chronic periodontitis: A randomized controlled trial.	Journal of Indian Society of Periodontology	16	2	179–83.	
148	Kranti, K., Seshan, H. & Sameer, Z.	2010	Clinical evaluation of topical subgingival application of biodegradable xanthan based 1.5% Chlorhexidine gel for treatment of periodontal pockets.	Journal of Advanced Oral Research	1	1	47–54	
149	Krück, C., Eick, S., Knöfler, G. U., Purschwitz, R. E. & Jentsch, H. F. R.	2012	Clinical and microbiologic results 12 months after scaling and root planing with different irrigation solutions in patients with moderate chronic periodontitis: A pilot randomized trial.	Journal of Periodontology	83	3	312–320	
150	Leiknes, T., Leknes, K. N., Böe, O. E., Skavland, R. J. & Lie, T.	2007	Topical use of a metronidazole gel in the treatment of sites with symptoms of recurring chronic inflammation.	Journal of Periodontology	78	8	1538–1544	
151	Luchesi, V. H., Pimentel, S. P., Kolbe, M. F., Ribeiro, F. V., Casarin, R. C., Nociti, F. H., Sallum, E. A., et al.	2013	Photodynamic therapy in the treatment of class II furcation: a randomized controlled clinical trial.	Journal of Clinical Periodontology	40	8	781–788	
152	Machion, L., Andia, D. C., Benatti, B. B., Carvalho, M.	2004	Locally delivered doxycycline as an	Journal of Periodontology	75	3	464–469	

	D., Nogueira-Filho, G. R., Casati, M. Z., Nociti, F. H., et al.		adjunctive therapy to scaling and root planing in the treatment of smokers: A clinical study.					
153	Malgikar, S., Reddy, Sh., Sagar, Sv., Satyanarayana, D., Reddy, Gv. & Josephin, Jj.	2016	Clinical effects of photodynamic and low-level laser therapies as an adjunct to scaling and root planing of chronic periodontitis: A split-mouth randomized controlled clinical trial.	Indian Journal of Dental Research	27	2	121–126	
154	Matesanz, P., Herrera, D., Echeverría, A., O'Connor, A., González, I., Sanz, M., O'Connor, A., et al.	2013	A randomized clinical trial on the clinical and microbiological efficacy of a xanthan gel with chlorhexidine for subgingival use.	Clinical Oral Investigations	17	1	55–66	
155	Paolantonio, M., D'Angelo, M., Grassi, R. F., Perinetti, G., Piccolomini, R., Pizzo, G., Annunziata, M., et al.	2008	Clinical and microbiologic effects of subgingival controlled-release delivery of chlorhexidine chip in the treatment of periodontitis: A multicenter study.	Journal of Periodontology	79	2	271–282	
156	Paolantonio, M., D'Ercole, S., Pilloni, A., D'Archivio, D., Lisanti, L., Graziani, F., Femminella, B., et al.	2009	Clinical, microbiologic, and biochemical effects of subgingival administration of a xanthan-based chlorhexidine gel in the treatment of periodontitis: A randomized multicenter trial.	Journal of Periodontology .	80	9	1479–1492	
157	Paolantonio, M., Dolci, M., Perfetti, G., Sammartino, G., D'Archivio, D., Spoto, G., Ciampoli, C., et al.	2008	Effect of a subgingival chlorhexidine chip on the clinical parameters and the levels of alkaline phosphatase activity in gingival	Journal of Biological Regulators and Homeostatic Agents	22	1	63–72	

			crevicular fluid during the non-surgical treatment of periodontitis.					
158	Reddy, S., Reddy, S. & Bhowmick, N.	2016	A comparison of chlorhexidine and tetracycline local drug delivery systems in management of persistent periodontal pockets - A clinical study.	International Journal of Applied Dental Sciences	2	3	11-15	
159	Sakellari, D., Ioannidis, I., Antoniadou, M., Slini, T. & Konstantinidis, A.	2010	Clinical and microbiological effects of adjunctive, locally delivered chlorhexidine on patients with chronic periodontitis.	Journal of the International Academy of Periodontology	12	1	20–26	
160	Schär, D., Ramseier, C. A., Eick, S., Mettraux, G., Salvi, G. E., & Sculean, A.	2020	Transgingival photodynamic therapy (tg-aPDT) adjunctive to subgingival mechanical instrumentation in supportive periodontal therapy. A randomized controlled clinical study.	Photodiagnosis and Photodynamic Therapy	32		101971	
161	Soeroso, Y., Soenarto, H., Bachtiar, B. M., Kemal, Y., Masulili, S. L. & Lessang, R.	2016	Efficacy of local minocycline HCl 2% gel as adjuvant for scaling and root planing in chronic periodontitis : A prospective randomized open blinded endpoint study	Current Issues in Periodontics			69–77	
162	Stelzel, M. & Florès-de-Jacoby, L.	2000	Topical metronidazole application as an adjunct to scaling and root planing.	Journal of Clinical Periodontology	27	6	447–452	

163	Tabenski, L., Moder, D., Cieplik, F., Schenke, F., Hiller, K. A., Buchalla, W, Schmalz, G. et al.	2017	Antimicrobial photodynamic therapy vs. local minocycline in addition to non-surgical therapy of deep periodontal pockets: A controlled randomized clinical trial.	Clinical Oral Investigations	21	7	2253-2264	
164	Theodoro, L. H., Silva, S. P., Pires, J. R., Soares, G. H. G., Pontes, A. E. F., Zuza, E. P., Spolidório, D. M. P., et al.	2010	Clinical and microbiological effects of photodynamic therapy associated with nonsurgical periodontal treatment. A 6-month follow-up.	Lasers in Medical Science	27	4	687–693	
165	Tomasi, C., Koutouzis, T. & Wennström, J. L.	2008	Locally delivered doxycycline as an adjunct to mechanical debridement at retreatment of periodontal pockets.	Journal of Periodontology	79	3	431–439	
166	Van Dyke, T. E., Offenbacher, S., Braswell, L. & Lessem, J.	2002	Enhancing the value of scaling and root-planing: Arestin clinical trial results.	Journal of the International Academy of Periodontology	4	3	72–76	
167	Williams, R. C., Paquette, D. W., Offenbacher, S., Adams, D. F., Armitage, G. C., Bray, K., Caton, J., et al.	2001	Treatment of periodontitis by local administration of minocycline microspheres: A controlled trial.	Journal of Periodontology	72	11	1535–1544	
168	Zhao, N.; Ge, S.H.; Ding, G.Y.	2006	Clinical effect of minocycline as adjunctive therapy to scaling and root planning on treatment of chronic periodontitis.	Hua Xi Kou Qiang Yi Xue Za Zhi [West China Journal of Stomatology]	24	1	32-35	

170	FDA Center for Drug Application Reviews Statistical Review and Evaluation	2001	Application Number NDA 50-781	https://www.accessdata.fda.gov/drugsatfda_docs/nda/2001/50781_Arestin_statr.pdf	

Table S2. Judgment of each risk of bias item for each included study according ROB2

Domain	Randomisation process		Deviations from intended interventions		Missing outcome data		Measurement of the outcome		Selection of the reported result		Overall Bias
Study	Authors' Judgment	Support for Judgment	Authors' Judgment	Support for Judgment	Authors' Judgment	Support for Judgment	Authors' Judgment	Support for Judgment	Authors' Judgment	Support for Judgment	
(Bogren et al. 2008)	Some concerns	Computer-generated permuted block; No information on concealment; no imbalances are apparent	Some concerns	Blinding of participant, clinician not reported; intention-to-treat analysis	Low	4/128 lost, ITT	Some concerns	Appropriate, blinding of examiner not reported	Low	Evidence of selective reporting was not detected	Some concerns
(Carvalho et al. 2007)	Some concerns	Coin toss; No information on concealment; no imbalances	Some concerns	Triple blinded; per protocol analysis	Low	2/28 lost (7.14%)	Low	Appropriate, blinded examiner	Low	Evidence of selective reporting was not	Some concerns

	Some concerns	are apparent								detected	
(Grisi et al. 2002)	Some concerns	No information on randomisation, concealment; no imbalances are apparent	High	Single blinded; per protocol analysis	Low	1/20 lost (5%)	Low	Appropriate, blinded examiner	Low	Evidence of selective reporting was not detected	High
(Harmouche et al. 2019)	Low	Computer-generated randomly permuted block; opaque envelopes; no imbalances are apparent	Some concerns	Sham used; per protocol analysis	High	8/36 lost (22.22%)	Low	Appropriate, blinded examiner	Low	Evidence of selective reporting was not detected	High
(Kasaj et al. 2007)	Some concerns	No information on randomisation, concealment; no imbalances are apparent	High	Single blinded; per protocol analysis	Low	0/20 lost	Low	Appropriate, blinded examiner	Some concerns	Adverse event not reported	High
(Katsikanis et al. 2020)	Some concerns	randomisation chart; No information on	Some concerns	Blinding of participant, clinician not	Low	0/21 lost	Low	Appropriate, blinded examiner	Low	Evidence of selective reporting	Some concerns

		concealment; no imbalances are apparent		reported; per protocol analysis						was not detected	
(Killeen et al. 2018)	Some concerns	Coin toss; No information on concealment; no imbalances are apparent	Some concerns	Blinding of participants, clinician not specified; per protocol analysis	Some concerns	7/55 lost (12.7%)	Low	Appropriate, blinded examiner	Low	Evidence of selective reporting was not detected	Some concerns
(Lulic et al. 2009)	Some concerns	randomisation table; No information on concealment;	Low	Sham used; per protocol analysis	Low	0/10 lost	Low	Appropriate, blinded examiner	Some concerns	Adverse event not reported	Some concerns
(Megally et al. 2020)	Some concerns	Computer-generated randomisation list; No information on concealment; no imbalances are apparent	Some concerns	Blinding of participant, clinician not reported; per protocol analysis	Low	0/32 lost	Low	Appropriate, blinded examiner	Low	Evidence of selective reporting was not detected	Some concerns
(Mizrak et al. 2006)	Some concerns	Coin toss; No information on concealment;	High	Single blinded; per protocol	Low	0/34 lost	Some concerns	Appropriate, blinding of examiner not	Some concerns	Adverse event not reported	High

		no imbalances are apparent		analysis				reported			
(Müller Campanile et al. 2015)	Low	Computer-generated table; serially numbered sealed envelopes; no	Some concerns	Sham used; per protocol analysis	Low	1/28 lost (3.6%)	Low	Appropriate, blinded examiner	Low	Evidence of selective reporting	Some concerns
(Petelin et al. 2015)	Some concerns	No information on randomisation, concealment; no imbalances are apparent	Some concerns	Blinding of participant, clinician not reported; per protocol analysis	Low	0/27 lost	Some concerns	Appropriate, blinding of examiner not reported	Some concerns	Adverse event not reported	Some concerns
(Shalev 2019)	Some concerns	No information on randomisation, concealment; no imbalances are apparent	Low	Sham and placebo used; intention-to-treat analysis	Low	1/59 lost, TTL	Low	Appropriate, blinded examiner	Some concerns	Adverse event not reported	Some concerns
(Sukumar et al. 2020)	Some concerns	Randomisation using sealed and opaque envelopes	High	Single blinded; per protocol analysis	Low	3/33 lost (9.1%)	Low	Appropriate, blinded examiner	Some concerns	Nonparametric data but report in mean	High

(van Steenberghe et al. 1999)	Some concerns	Computer-generated randomisation code; No information on concealment; no imbalances are apparent	Some concerns	Placebo used; per protocol analysis	Some concerns	11/93 lost (11.83%)	Some concerns	Appropriate, blinding of examiner not reported	Low	Evidence of selective reporting was not detected	Some concerns
(Kessler et al. 2021)	Some concerns	Computer-generated randomisation table; No information on concealment; no imbalances are apparent	High	Single blinded; per protocol analysis	High	5/22 lost (22.7%)	Some concerns	Appropriate, blinding of examiner not reported	Some concerns	Data presented in graphs rather than raw data	High

**Overall risk-of-bias judgement**	**Criteria**
**Low risk of bias**	The study is judged to be at low risk of bias for all domains for this result.
**Some concerns**	The study is judged to raise some concerns in at least one domain for this result, but not to be at high risk of bias for any domain.
**High risk of bias**	The study is judged to be at high risk of bias in at least one domain for this result. Or<br>The study is judged to have some concerns for multiple domains in a way that substantially lowers confidence in the

	result.
